# Re-engineering alum sludge using choline–glycine ionic liquid for enhanced dewatering and dye adsorption in wastewater treatment

**DOI:** 10.1038/s41598-026-65696-x

**Published:** 2026-08-24

**Authors:** Aya M. Rashed, Ibrahim E. T. El-Sayed, Xingmei Lu, Hamed M. Abdel-Bary, Mai K. Fouad, Maha A. Tony

**Affiliations:** 1 Advanced Materials/Solar Energy and Environmental Sustainability (AMSEES) Laboratory, Basic Engineering Science Department, Faculty of Engineering, Menoufia University, Shebin El-Kom, Egypt; 2 Chemistry department, Faculty of Science, Menoufia University, Shebin El-Kom, Egypt; 3 Beijing Key Laboratory of Solid-State Battery and Energy Storage Process, CAS Key Laboratory of Green Process and Engineering, Institute of Process Engineering, Chinese Academy of Sciences, Beijing, 100190 China; 4 School of Chemical Engineering, University of Chinese Academy of Sciences, Beijing, 100049 China; 5 Chemical Engineering Department, Faculty of Engineering, Cairo University, Giza, Egypt; 6 Planning and Construction of Smart Cities Program, Faculty of Engineering, Menoufia National University, Menoufia, 32651 Egypt

**Keywords:** Adsorption, Alum sludge, Conditioning, Dewatering, Ionic liquid, Textile dyes, Chemistry, Engineering, Environmental sciences, Materials science

## Abstract

Large quantities of by-product alum sludge (Al-S) generated from drinking water treatment plants pose significant environmental and disposal challenges due to their high water content and large volume. This study aims to develop a sustainable conditioning and valorization strategy for Al-S using a choline–glycine ionic liquid ([Ch]–[AA]). The ionic liquid was synthesized and applied as a sludge conditioner to enhance dewatering performance. Under optimized conditions, the dewatering efficiency reached 47%, accompanied by a notable zeta potential shift, indicating partial charge neutralization and improved floc structure. To enable sludge valorization, the conditioned sludge was reused as an adsorbent, either directly or after thermal treatment. The modified materials exhibited high adsorption capacities toward Synozol KHL dyes, reaching 43.6 and 90.1 mg g⁻^1^ for Red and Blue dyes, respectively. Adsorption behavior was well described by the Langmuir isotherm and pseudo-second-order kinetic model. Thermodynamic analysis indicated an exothermic adsorption process, with spontaneity dependent on operating conditions. Overall, the results demonstrate an effective waste-to-resource approach in which enhanced dewatering facilitates the conversion of alum sludge into a functional adsorbent for wastewater treatment, supporting sustainable sludge management.

## Introduction

Rapid urbanization, population growth, and changing lifestyles have significantly increased the demand for potable water^1,2^. From an engineering perspective, the production of safe drinking water inevitably leads to the generation of waterworks residuals^3–5^. Drinking water treatment plants produce large quantities of alum sludge (*Al-S*) as a by-product of using aluminum sulphate during coagulation and flocculation processes^6–8^, where metal-based coagulants are applied to destabilize and aggregate suspended particles and colloidal impurities, facilitating their removal through flocculation and sedimentation^9^. Annually, a single treatment plant can generate hundreds of thousands of tons of *Al-S*, while global sludge production exceeds 10,000 tons per day^10–12^. However, global alum sludge production remains only roughly estimated^13^.

The physicochemical properties of *Al-S* vary depending on the coagulant used and the characteristics of the treated raw water, particularly its turbidity and solids content^14,15^. Consequently, *Al-S* exhibits highly variable solids content but is universally classified as a “difficult-to-dewater” material^13,16^. Conventional disposal practices include discharge into natural water bodies, sewer systems, or landfilling^17^. Although economically attractive, these approaches pose severe environmental risks, including soil and water contamination, and are no longer considered sustainable solutions^6,18,19^. As a result, environmentally friendly and efficient *Al-S* management strategies remain major challenge for both industry and academia^20^.

Increasingly stringent environmental regulations emphasize the need for sustainable sludge management practices^21–23^. Typically, *Al-S* management involves successive steps, including thickening, stabilization, dewatering, and final disposal, with dewatering identified as a critical operation for volume reduction^3,15^. To enhance sludge dewaterability, conditioning represents an essential pretreatment step^24–26^, as it reduces water content and overall sludge volume^27^. Traditionally, *Al-S* conditioning has relied on chemical additives such as flocculants, acids, ferric chloride, and lime^28,29^, as well as physical disruption techniques^30^. While chemical conditioners, including polyelectrolytes, have demonstrated superior dewatering efficiency^31–34^, their associated toxicity raises environmental concerns. Recently, ionic liquids (ILs) have emerged as promising alternatives to conventional conditioners^35^. However, despite their potential advantages, few studies have investigated ILs for sludge conditioning^36^, and to the best of the authors’ knowledge, no previous work has addressed the application of ILs for alum sludge conditioning^37,38^. Consequently, significant research gaps remain regarding the role of ILs in enhancing sludge dewaterability and the associated mechanisms.

Choline–amino acid ionic liquids ([Ch]-[AA]) have recently emerged as promising alternatives to conventional sludge conditioning agents due to their tunable structure, biocompatibility, and low toxicity^24–27,37^. Unlike traditional polyelectrolytes, which primarily rely on charge neutralization and bridging mechanisms, [Ch]-[AA] ionic liquids provide combined electrostatic interactions, hydrogen bonding, and complexation capabilities through their cation–anion pairs and functional groups such as –COO⁻ and –NH₂^2,35^. These features enable more effective interaction with alum sludge components, including amorphous aluminum hydroxides and organic matter, promoting floc restructuring and enhanced aggregation. In addition, the amphiphilic nature of such ionic liquids can facilitate bound water release and modification of extracellular polymeric substances (EPS), further improving dewatering performance^38,39^. Therefore, [Ch]-[AA] ionic liquids offer a mechanistically distinct and potentially more sustainable approach for sludge conditioning compared with conventional chemical additives.

It is hypothesized that the choline–glycine ionic liquid enhances sludge conditioning through partial surface charge neutralization and compression of the electrical double layer, thereby reducing interparticle repulsion^34^. In addition, the presence of functional groups such as –COO⁻ and –NH₂ is expected to facilitate hydrogen bonding, complexation, and bridging interactions, promoting floc aggregation and structural reorganization^35,38^. Furthermore, these functionalities may introduce active adsorption sites on the conditioned sludge surface, thereby improving its affinity toward dye molecules during subsequent valorization^17^.

Herein, an integrative strategy for alum sludge (Al-S) management is proposed through the application of a choline–glycine amino acid-based ionic liquid as a dual-functional material. To the best of our knowledge, studies on the application of biodegradable amino acid-based ionic liquids for alum sludge conditioning remain limited. The proposed approach combines sludge conditioning with subsequent sludge valorization, providing an integrated pathway for waste minimization and resource recovery that supports the principles of the circular economy.

The objectives of this study were threefold. First, to investigate the effectiveness of choline–glycine ionic liquid as a green conditioning agent for improving alum sludge dewaterability. Second, to elucidate the mechanisms responsible for dewatering enhancement through physicochemical characterization of the conditioned sludge. Finally, to evaluate the feasibility of converting the conditioned sludge into an adsorbent for the removal of Synozol KHL Red and Synozol KHL Blue dyes from wastewater.

## Experimental section

### Preparation of the ionic liquid

An alternative synthesis route for the choline–glycine ionic liquid ([Ch]-[AA]) was performed using commercially available choline chloride instead of choline hydroxide. Choline chloride (≥ 99%), glycine (≥ 98.5%), and potassium hydroxide (KOH, ≥ 85%) were purchased from Sigma-Aldrich. Ethanol (95%) was used as a solvent and purchased from a certified local supplier. All chemicals were of analytical grade and used without further purification.

The ionic liquid was synthesized by adding an ethanolic solution of potassium hydroxide (1 mmol) to a mixture of choline chloride (1 mmol) and glycine (1.05 mmol) under continuous stirring for 72 h at room temperature. After completion of the reaction, the mixture was filtered to remove the insoluble potassium chloride by-product. The solvent (ethanol) was subsequently removed by rotary evaporation under reduced pressure for 3 h, followed by drying to obtain the final product.

**Scheme 1:**
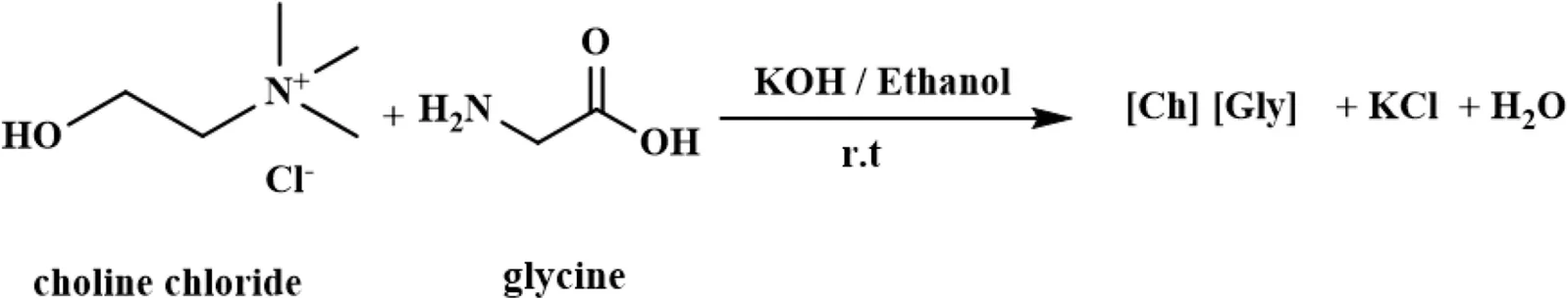
Alternative pathway for synthesis of ionic liquid choline based – Amino acids.

**Fig. 1:**
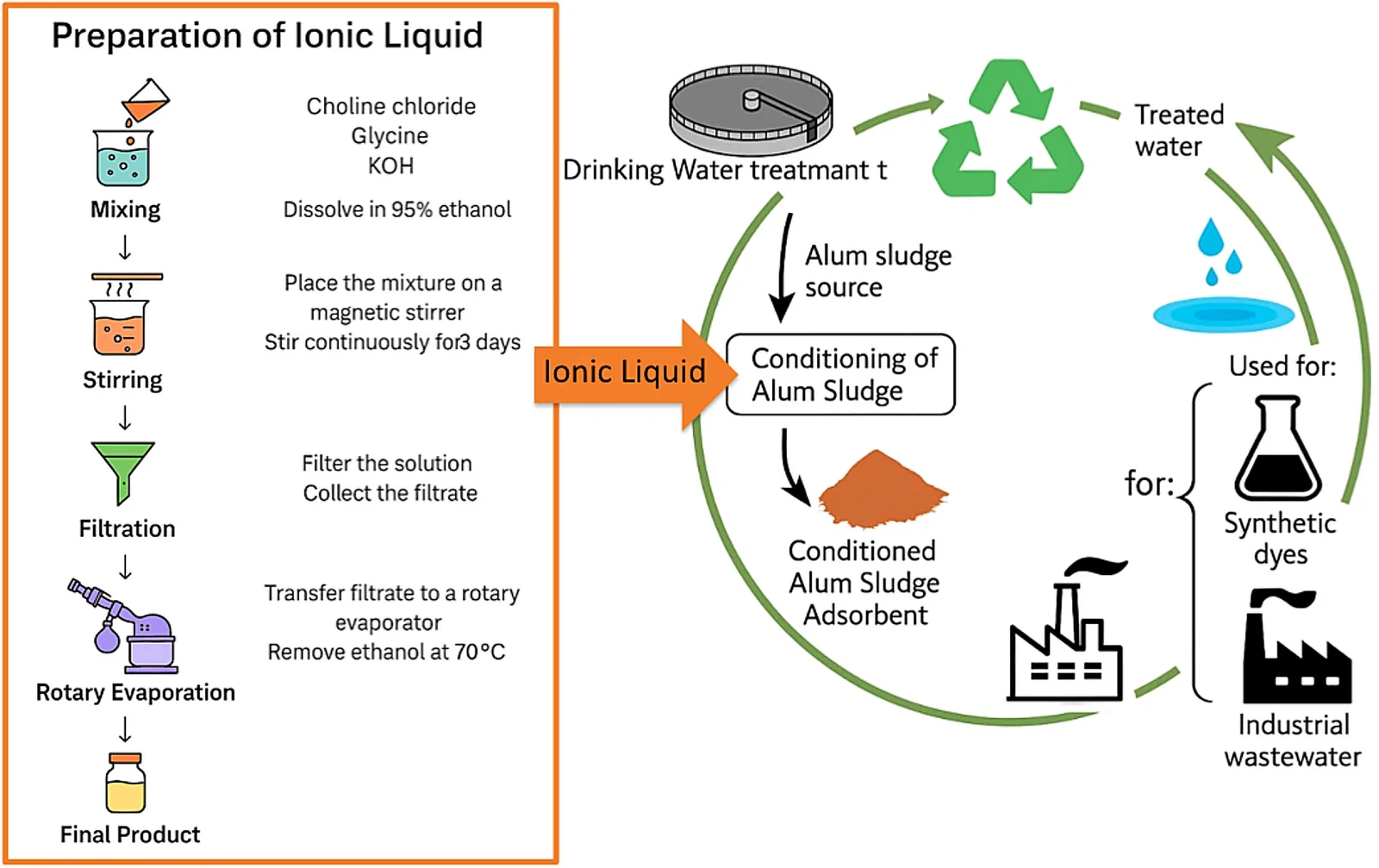
Schematic representation of ionic liquid conditioner preparation and wastewater treatment using conditioned alum sludge.

The synthesized ionic liquid was obtained in a yield of 11.74 g and a purity of 92%, as confirmed by FTIR and NMR analyses. The density of the synthesized choline–glycine ionic liquid was assumed to be approximately 1.0 g mL⁻^1^, consistent with reported values for similar amino acid–based ionic liquids^40^. The synthesis procedure is schematically illustrated in Scheme 1, and the preparation steps are summarized in Fig. 1.

### *Alum sludge* sampling

Alum sludge (Al-S) was collected from a drinking water treatment plant located in Shebin El-Kom City, Menoufia Governorate, Egypt, where aluminum sulphate is used as the primary coagulant. The collected sludge was immediately transferred to the laboratory in clean, acid-washed plastic containers and stored at 4 °C prior to analysis, following standard methods. The conditioning and handling procedures are schematically illustrated in Fig. 1. The physicochemical characteristics of the raw *Al-S* are summarized in Table 1. The initial properties included a capillary suction time (CST) of 44 s, total suspended solids of 16,015 mg L⁻^1^, pH 7.5, supernatant turbidity of 4.9 NTU, and a specific resistance to filtration (SRF) of 2.59 × 10^11^ m kg⁻^1^.

**Table 1:** The main physicochemical characteristics of raw alum sludge and its supernatant.

Parameters	Unit	Value
Suspend solid	mg L^-1^	16,015
pH	-	7.5
SRF	m kg^-1^	2.59 × 10^11^
CST	s	44.0
Supernatant		
Turbidity	NTU	4.90
Fe	mg L^-1^	0.38
Mn	mg L^-1^	0.20
phosphate ion (PO_4_^3^⁻)	mg L^-1^	0.17
sulfate ion (SO_4_^2^⁻)	mg L^-1^	33.0
ammonium ion (NH_4_⁺ )	mg L^-1^	14.21
Al	mg L^-1^	0.30

### Wastewater

Synthetic dyes, Synozol KHL Red and Synozol KHL Blue, were used as model water-soluble reactive dyes to simulate textile wastewater. Both dyes were supplied by Kisco International. Separate stock solutions (1000 mg L⁻^1^) were prepared for each dye using deionized water and subsequently diluted to the desired concentrations for experimental use.

### Methodology

Primarily, 100 mL of Al-S samples were transferred to a beaker for jar test experiments. The pH was adjusted, where necessary, to the desired values using diluted sulphuric acid or sodium hydroxide solutions. The sludge conditioning experiments were conducted at room temperature (25 ± 2 °C), while additional temperature-controlled experiments were performed within the range of 25–80 °C to evaluate the influence of thermal conditions on process performance. The sludge samples were conditioned by adding the ionic liquid at dosages ranging from 0.005 to 0.02 mL L⁻^1^, corresponding to an actual addition of 0.0005–0.002 mL per 100 mL sample. The applied volumetric dosages were converted into mass-based units (mg L⁻^1^) using an assumed ionic liquid density of approximately 1.0 g mL⁻^1^, and subsequently normalized to the sludge dry solids content, expressed as mg g⁻^1^ dry solids (DS). Accordingly, the tested range corresponds to approximately 0.31–1.25 mg g⁻^1^-DS. Following the addition of the ionic liquid (or other conditioners), the samples were subjected to rapid stirring (200 rpm) for 30 s to ensure homogeneous dispersion, followed by slow mixing (40 rpm) for the designated reaction time (1–12 min) in a jar test apparatus to promote flocculation and enhance sludge conditioning.

To evaluate the sustainability trend of the conditioned sludge with green ionic liquid, the sludge material was subsequently evaluated for the adsorption process. The conditioned sludge was air dried before being oven dried for 1 h (105ºC). Afterwards, the dried material was then grinded in mortar and pestle and the resultant material is used as adsorbent and refereed to as *Al-S* /IL. Subsequently, *Al-S* /IL is then calcined in an electrical furnace for 2 h at different temperatures with a rate of 10 ºC/min at 400, 600 or 800ºC and the resulting material were labeled as *Al-S* /IL400, *Al-S* /IL600, and *Al-S* /IL800.

### Additional conditioner

Further organic and inorganic conditioners were applied to aluminum sludge (Al-S) to enhance its dewaterability and to enable comparison with the studied ionic liquid conditioner. An anionic surfactant, sodium dodecyl sulfate (SDS, Sigma-Aldrich; molecular weight 288.38 g mol⁻^1^), was used as a representative organic conditioner to improve flocculation and dewatering performance. SDS was applied at a concentration of 50 mg L⁻^1^. In addition, an inorganic conditioner, gypsum (CaSO₄·2H₂O, calcium sulfate dihydrate; supplied by Saint-Gobain Gyproc Egypt), was employed as a skeleton builder to improve sludge structure and permeability. The material has a density of approximately 1,400 kg m⁻^3^ and was evaluated in comparison with organic conditioners. Moreover, organic polymeric conditioners of different charge types were investigated. An anionic polymer, Magnafloc LT-25 (CIBA Specialty Chemicals Ltd., UK; molecular weight 10–15 × 10⁶), and a cationic polymer, Magnafloc FO-4140 (SNF SAC, France; molecular weight ~ 5 × 10⁶), were used. Polymer stock solutions were prepared at a concentration of 0.01% (w/v) and applied at specific dosages. Additionally, Fenton-type oxidation was employed as a comparative conditioning approach using hydrogen peroxide (H₂O₂, 30%, Sigma-Aldrich) and ferric sulfate (Fe₂ (SO₄)₃·H₂O; molecular weight 399.88 g mol⁻^1^, QualiKems) as a catalyst at pH 3.0 to ensure a consistent comparison basis.

### Adsorption studies

Batch adsorption experiments were conducted to evaluate the performance of the prepared adsorbents under controlled conditions. All experiments were carried out at room temperature (25 ± 2 °C). The adsorption behavior was evaluated as a function of contact time, initial dye concentration, adsorbent dosage, pH, and temperature. The contact time was varied until equilibrium was reached, which was reached after approximately 5 h for Synozol KHL Red and 24 h for Synozol KHL Blue, based on the adsorption time-profile results. Kinetic studies were performed by collecting samples at selected time intervals throughout the adsorption process to monitor the dye removal process. For isotherm analysis, the initial dye concentration was varied within the range of 10–100 mg L⁻^1^, while maintaining constant adsorbent dosage and solution conditions. Adsorbent dosage was examined in the range of 0.5–2.0 g L⁻^1^, and pH was adjusted between 2 and 10 to evaluate its influence on adsorption performance. Temperature-dependent studies were conducted over the range of 25–80 °C to assess thermodynamic behavior. After adsorption, samples were filtered and analyzed using UV–Vis spectrophotometry to determine residual dye concentrations. To ensure experimental reproducibility, each experiment was independently repeated three times under identical conditions, and the reported results correspond to the average of the three measurements.

### Alum sludge and wastewater analytical assessment

Dewatering efficiency was calculated based on the reduction in capillary suction time (CST) relative to the raw sludge (%CST = [(CST₀ − CST)/CST₀] × 100). Specific resistance to filtration (SRF) and moisture content were used as complementary indicators to further evaluate sludge dewaterability. CST was measured using a Triton CST apparatus, while SRF was determined following previously reported procedures^22^. Moisture content was evaluated according to Standard Methods for the Examination of Water and Wastewater.

The characteristics of the sludge supernatant before and after conditioning were also evaluated. Total dissolved solids (TDS) were measured using a HACH HQ40d meter, turbidity using a HACH 2100Q turbidimeter (Lovibond TB350 IR), and zeta potential using a Zetasizer (Malvern Zetasizer Nano ZS, Anton Paar Litesizer 500). Chemical oxygen demand (COD) was determined using a HACH DRB200/DR3900 system, viscosity using an Anton Paar Rheolab QC viscometer, and pH using a digital pH meter (AD1030, Adwa Instruments, Hungary).

After wastewater treatment, samples were periodically collected at predetermined time intervals and subjected to microfiltration prior to spectrophotometric analysis. The residual concentrations of Synozol KHL Red and Synozol KHL Blue dyes in the aqueous phase were determined using a UV–Vis spectrophotometer (Unico UV-2100, USA).

All experiments were conducted in three triplicates, and the reported values represent the mean ± standard deviation.

### Characterization

X-ray diffraction (XRD) was used to investigate the structure of the synthesized ionic liquid, conditioned sludge and treated sludge derived adsorbent composite. Bruker-Nonius Kappa CCD diffractometer, which is equipped with CuKα radiation at a wavelength of 1.5406 Å was used to obtain the XRD pattern of the materials. Also, C13-NMR (Carbon-13 Nuclear Magnetic Resonance) and NMR analyses were conducted using Bruker Avance III HD 400 MHz, Jeol ECZ 400S NMR spectroscopy for assessing the prepared ionic liquid. Moreover, the morphology of the prepared ionic liquid or alum sludge before and after conditioner as well as the treated absorbent sludge composite were imaged by Field-emission scanning electron microscope (SEM) image using FE-SEM, Quanta FEG 250. Such instrument is augmented with energy dispersive spectrum X-ray spectroscopy (EDX) to examine the main oxides contained in raw and conditioned sludge were analyzed. Additionally, the particle size distribution was assessed using imageJ 1.48 V program. Fourier transform infrared FTIR spectra (Jasco, FT/IR-4100, type A) of the raw alum sludge, conditioned sludge or for ionic liquid were conducted to identify the functional group of such materials. Zeta potential (ζ-potential) of alum sludge was determined using a Zetasizer (Zetasizer Ver. 6.32, Malvern Instrument Ltd., Worcestershire, United Kingdom) at 25 °C.

## Results and discussion

### Characterization of choline glycine IL

#### FTIR analysis

**Fig. 2:**
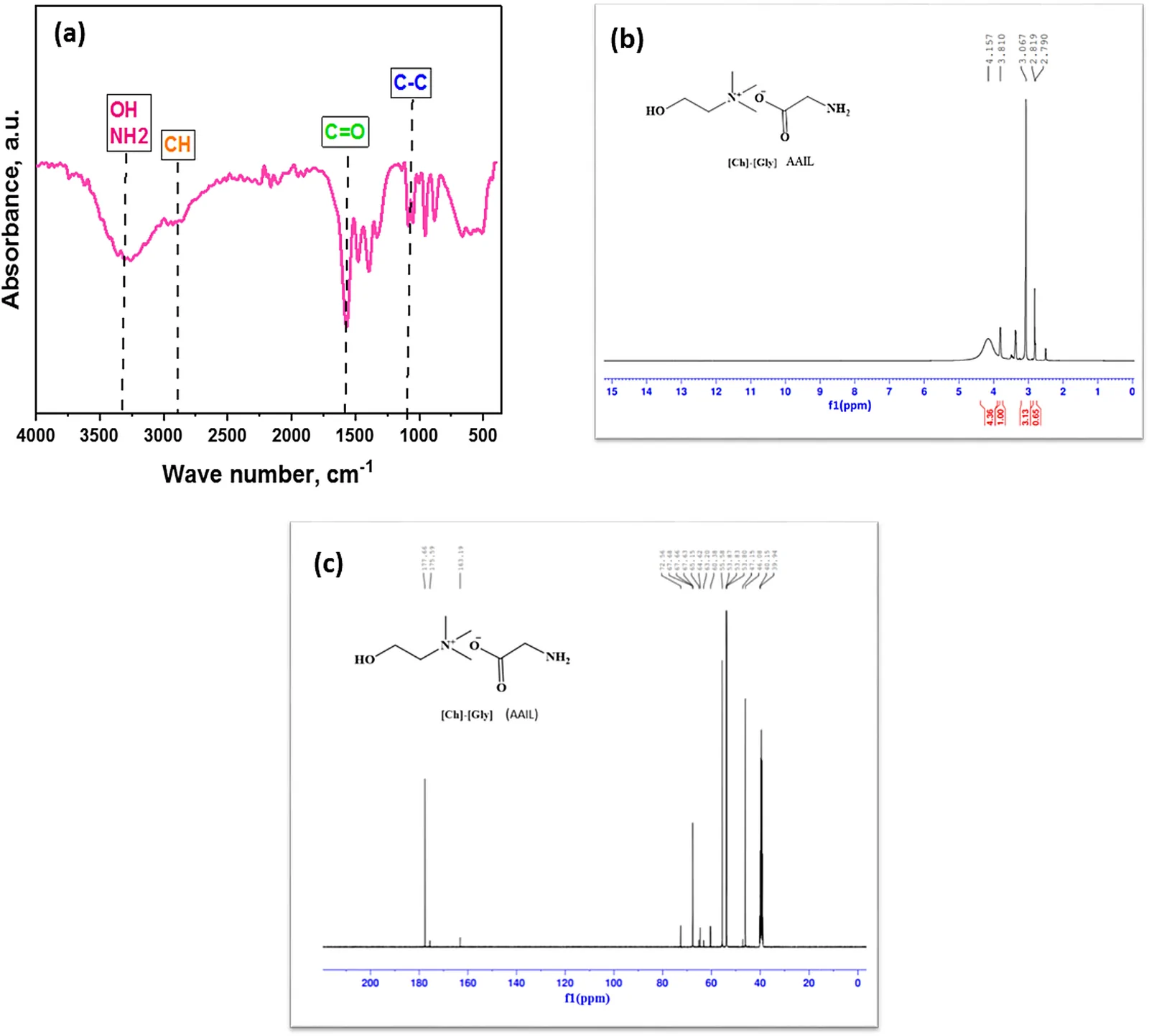
Characterization of Choline Glycine IL (Amino Acid Ionic liquid (AAIL) (**a**) FTIR, (**b**) C13 NMR and (**c**) H1 NMR.

The FTIR spectrum of [Ch][Gly] ionic liquid was evaluated and the data are shown in Fig. 2 (a). The results exhibited a broad absorption band at 3276 cm⁻^1^, which arises from the overlapping stretching vibrations of hydroxyl (O–H) and amino (N–H) groups, indicating the coexistence of the choline hydroxyl moiety and the primary amine of the glycinate anion. The peak observed at 2965 cm⁻^1^ corresponds to aliphatic C–H stretching vibrations from the methyl and methylene groups in both the cation and the anion. A distinct band at 1573 cm⁻^1^ is assigned to the asymmetric stretching of the carboxylate group (ν_asCOO⁻), confirming the deprotonation of the carboxylic acid functionality and the formation of the glycinate anion. The band at 1208 cm⁻^1^ is attributed to C–N and C–O stretching vibrations within the choline backbone. Collectively, these results confirm the ionic nature of the compound and the successful synthesis of the choline glycinate ionic liquid^40^.

#### ^13^C-NMR

The ^13^C-NMR spectrum (101 MHz, D₂O) of [Ch][Gly] reveals seven distinct resonances as shown in Fig. 2 (b). The signal at δ 177.53 ppm corresponds to the carboxylate carbon of the glycinate anion, confirming its deprotonated state. Signals at δ 72.42 ppm and δ 67.53 ppm are attributed to the methylene carbons adjacent to the hydroxyl group in the choline cation. Resonances at δ 55.45 ppm, δ 53.74 ppm, and δ 53.70 ppm correspond to the three methyl carbons bound to the quaternary nitrogen and the methylene carbon adjacent to nitrogen in the choline moiety. The peak at δ 45.95 ppm is assigned to the glycinate methylene carbon situated between the amino and carboxylate groups. The chemical shift distribution is fully consistent with the proposed ionic liquid structure and supports the FT-IR and ^1^H-NMR findings^41,42^.

#### ^1^H-NMR

The ^1^H-NMR spectrum (400 MHz, D₂O) of [Ch][Gly] was recorded and the data shown in Fig. 2 (c) displays a singlet at δ 3.07 ppm integrating for nine protons, corresponding to the three equivalent methyl groups bound to the quaternary nitrogen atom in the choline cation. Multiplets at δ 3.81 ppm and δ 3.37 ppm are assigned to the methylene protons adjacent to the hydroxyl group (–CH₂–O–) and to the nitrogen atom (–CH₂–N⁺), respectively, within the choline moiety. The glycinate anion contributes a broad multiplet at δ 2.82 ppm, which is attributed to the methylene protons located between the amino and carboxylate groups (–CH₂–NH₂). A broad singlet at δ 4.36 ppm represents exchangeable protons from hydroxyl and amino groups, broadened due to proton exchange in D₂O. The chemical shift pattern and integration values were in full agreement with the expected structure of choline glycinate^40,43^.

### *Al-S* conditioning and dewatering performances

#### Conditioning-time based on IL conditioner

**Fig. 3:**
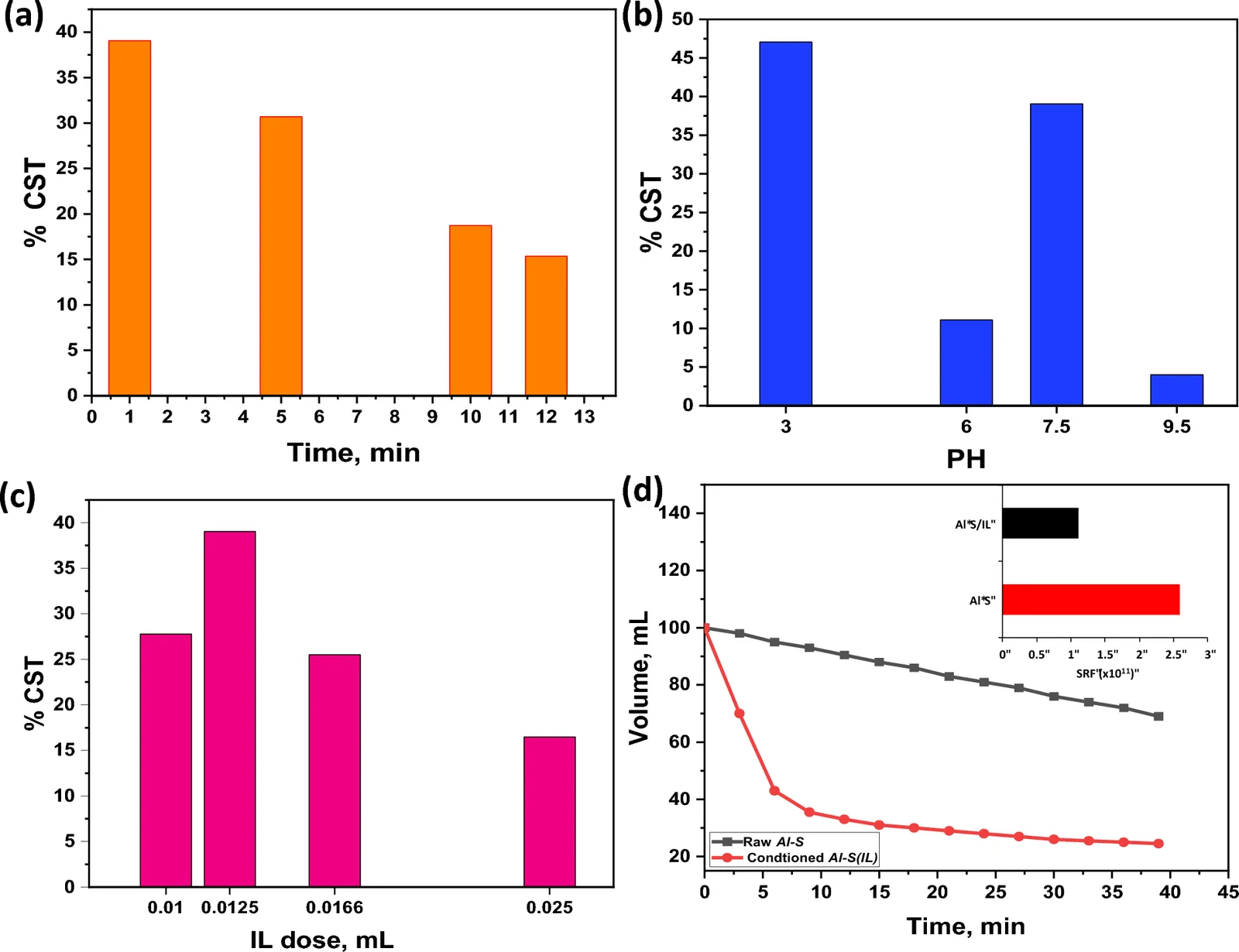
Effectiveness of alum sludge conditioning with ionic liquid on the sludge CST: (**a**) conditioning time; (**b**) pH effectiveness, (**b**) ionic liquid dose and (**d**) the corresponding settling test (inset SRF data).

*Al-S* was treated with IL as a green conditioner and conditioning time is illustrated together with the conditioner effectiveness in Fig. 3 (a). The effect of the IL conditioner on sludge dewatering is exhibited as evakby the significance of CST deduction. The experiment was conducted by changing the IL-conditioning time whereas all other operational parameters are kept constant, i.e. pH 3.0 and IL 0.00125 mL L^-1^. Generally, a lower CST indicates to the better alum sludge filterability. The effect of the conditioning test time on *Al-S* based sludge conditioning to enhance its dewatering performance were evaluated at varied flocculation time in the range of 1 to 12 min of flocculation time began with fast (30 s) stirring thereby followed by slow mixing at the specific reaction time to assure flocculation. The results displayed in Fig. 3 exhibited that the IL conditioner could result in an enhancement in the CST reduction. However, the maximal CST reduction (%) is corresponding to a specific time of conditioning that is 1 min of dewatering time.

The result showed that the IL effectively improved sludge conditioning and improved at minimal contact time of 1 min. But, sludge conditioning with the excess conditioning time more than 1 min is not favorable. This is due to the dewaterability capability that is signified by CST reduction is not enhanced. The CST reduction showed the highest dewatering performance at one minute in contact with IL to record about 40% CST reduction. Such investigation is in agreement with the previous research reported by^31^. This may be attributed to changes in floc size formed after adding flocculent material that is the main factor responsible for enhancing the dewaterability. Notably, to mention that the *Al-S* dewaterability improvement by the IL treatment dependent on the release of both interstitial and chemically bound water.

Furthermore, the comparative data of the settling properties is conducted to compare the settling tendency of the raw *Al-S* and the conditioned one with ionic liquid. The result showed in (d), that exhibit the position of the *Al-S* /supernatant interface that is correlated as a role of the time of settling that is up to 40 min. It can be observed from Fig. 3 (d) that the values of the settling behavior of *Al-S* /supernatant interface differ for the conditioned sludge than the raw *Al-S* with no conditioner with notable significant alteration. This confirms that the number and size of *Al-S* flocs after conditioning is enlarged in comparison to the raw sludge that the verifies the interstitial water is removed and the sludge become more compact and settled. Furthermore, the SRF is measured before and after IL conditioning and the data verified the role of ionic liquid since the SRF value is reduced from 2.59 × 10^11^ m kg^-1^ to 1.11 × 10^11^ m kg^-1^ after conditioning as exhibited in the inset of Fig. 3 (d). Thus, the IL reduced the water content that evaluated by specific resistance for filtration^31^.

#### Effect of *Al-S* pH

pH plays a vital role in substance hydrolysis, through acidic or alkaline circumstances since the colloidal particles of *Al-S* is affected with the pH value. Extracellular polyelectrolytes in the *Al-S* might be desecrated with the release of the interior water molecules from the *Al-S*. Large flocs may be undeniably created through the IL conditioning system. However, the charge conflict between the *Al-S* molecules via acidic system is larger than that under alkaline medium. In this regard, the pH value of the medium is altered from acidic to alkaline range. It is clear from Fig. 3 (b) that although the acidic and neutral pH are favorable, the acidic pH (3.0) results in a higher CST reduction (47%) compared to 39% when the pH is adjusted as neutral natural pH of the sludge. But, pH recorded at 5.0 or pH alkaline (9.5) recorded lower CST reduction efficiency. This could be attributed to the metal extraction by protic ionic liquids (i.e. [Hmim] Cl) that is significantly alters the availability and speciation of metals in *Al-S*. such specific pH values results in is high extraction of Al^3^⁺, Fe^3^⁺, and other trace elements without some other metals loss^44^. For instance, at acidic pH (~ 3.0), soluble multivalent cations remain available to interact electrostatically with ionic liquid anions, promoting robust floc bridging thereby this is leading to minimal CST values that results in a high CST reduction (%)^15,45^. However, pH in the range of 5.5–6.0, leading to the presence of metal hydroxide formation destabilizes surface charge and floc structure, thereby this is causing dispersion of particles and CST reduction in declined^46^. However, when the pH approaches neutrality (~ 7.5), partial flocculation resumes via Ca^2^⁺/Mg^2^⁺ bridging and residual IL interactions, moderately lowering CST. In strongly alkaline conditions (> 8.5), precipitation and strong negative surface charge prevent floc formation, causing CST to rise sharply^47^.

#### Effect of IL conditioner dose

Various sets of experiments at different IL conditioner concentrations were assessed at the optimal conditioning time of one minute using the IL dose from 0.01 to 0.025 mLL^-1^. The data obviously displayed in Fig. 3 (c), adding the ionic liquid dose enhanced the CST reduction to 27% when 0.01 mLL^-1^ is added and thereby it is increased to 39% when 0.0125 mL L^-1^ is added. The CST reduction demonstrates that the maximum CST reduction (%) is corresponded to a specific concentration of conditioning at 0.0125 mL L^-1^ that recorded 39% CST reduction. However, further increase in ionic liquid dosage 0.016 and 0.005 mL L^-1^ results in a decline of CST reduction % to 25 and 16%, respectively. Hence, an optimum dosage of 0.78 mg g⁻^1^-DS (0.0125 mL L⁻^1^) was identified, at which the highest dewatering performance was achieved (e.g., maximum reduction in [insert metric: CST/SRF/moisture content]). Below this dosage, insufficient conditioning limited flocculation efficiency, whereas higher dosages did not result in further improvement and may be attributed to restabilization of particles or saturation of available binding sites.

It is consistent to mention that the *Al-S* dewaterability improved by IL dependent on the release of both interstitial and chemically bound water. Interstitial water that is trapped between the organics and chemically bound water or adsorbed by the oxidation and destruction of organics. Notably. Such investigation is in agreement with the previous study reported in literature^32^. Since similar results have been reported regarding sludge dewatering ability improvement with the change in the conditioner dose. This could be associated with the presence of the bound water on the sludge. Bound water controls sludge dewaterability, and lessening such bound water content of *Al-S* improves its dewaterability performance. Further, elevating the IL dose affects the charge neutralization capability of *Al-S*, leaving more water bound to *Al-S* flocs. However, increasing the IL dose higher than such optimal dose might result in a decline in the sludge dewaterability and had a negative influence and weaken the dewatering performance. This could be linked to the role of ionic liquid in chemical structure of the *Al-S.* Some metals in *Al-S* were dissolved in ionic liquids during the conditioning process. Such investigation is in accordance with the previous work reported in literature^31^ in treating activated sludge by ionic liquid to improve its dewaterability.

### Comparison of sludge dewaterability by various conditioners

**Fig. 4:**
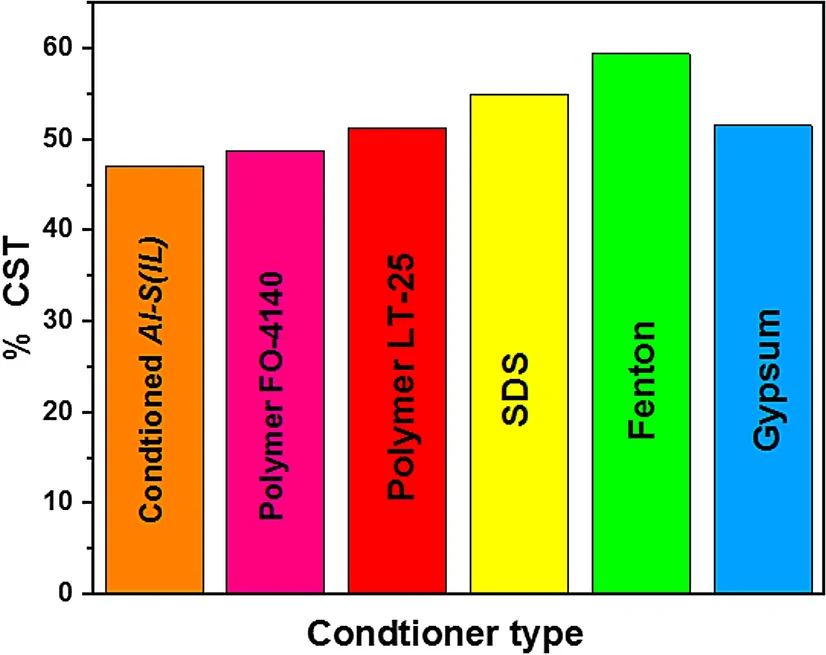
Effects of different conditioner on *Al-S* dewatering performance.

Generally, chemical based conditioners improve *AL-S* dewaterability by different mechanisms. In order to investigate the comparative data of suggested IL conditioning system with the traditional conditioners, the influence of numerous conditioners namely polymer conditioners such as FO-4140 and anionic LT-25 polyelectrolytes as well as anionic sodium dodecyl sulphate (SDS) surfactant is assessed to assess such dewaterability performance on *AL-S*. Figure 4 depicts typical data for the conditioning performance via CST reduction. According to the data exhibited in Fig. 4, it is clear that both polyelectrolytes have positive influence on the overall sludge filterability criteria, but the influence of both polyelectrolytes gets different CST correlations. Also, the anionic magnafloc LT-25 polyelectrolyte represents higher pronounced influence (51% CST reduction) than cationic FO-4140 one on the CST reduction (48%) achievement. The water content of the conditioned sludge is slightly declined by the selected polyelectrolytes type. Also, extra upsurge in the CST reduction (54%) SDS dose results in a higher pronounced influence in the CST reduction efficacy. The same trend is achieved when Fenton and gypsum are added. It is noteworthy to mention that the danger in adding toxic polyelectrolyte or surfactant conditioner is not merely the extra conditioning cost in reagents, but also the toxic discharge with not excess pronounced effective.

Polymer conditioning is leading to a decline in the tendency of water content of the dewatered *Al-S.* Such trend is achieved slightly following polymer addition on the *Al-S* due to the floc creation and the distinction in the *Al-S* ‘ cake porosity^48,49^. Additionally, the *Al-S* cell walls could be destroyed after both types of polymer addition that is then followed by the water release inside the cells. It is significant to mention that the FO-4140 polymer contains positively charged hydrophilic groups that perform a severe role in the hydrophobicity of the dewatered *Al-S* surface. But, LT-25 possesses a negatively charge hydrophilic groups that developed an adverse effect on the dewatering efficiency of *Al-S*. Hence, the dewaterability of the *Al-S* after LT-25 conditioning was consequently superior. Further, comparison of the CST as a dewaterability indicator of *Al-S* using SDS with that of the IL, the data depicted in Fig. 4 exposes the enhancement of dewaterability when SDS is applied since it reached 51%. Such data verifies that the volume of dewatered *Al-S* is reduced since the SDS enhances the dry solid content attains the floc creation. Notably, in such case the formed *Al-S* flocs in the intermediate are too minor in size, loose and fragile. This might be attributed to SDS is classified as amphiphilic organic compounds; thereby such substance has hydrophobic as well as hydrophilic groups. SDS might be absorbed onto the *Al-S* solid’s surface via different ion exchange and ion pairing besides hydrogen-bonding. Also, the presence of Van der Waals force and hydrophobic attachment prior to it exceeds the critical limits.

Furthermore, inorganic conditioners such as gypsum (CaSO_4_) as well as Ferric sulphate (FeSO_4_) that is a source of Fenton reaction conditioning system are also investigated. According to the data in Fig. 4, inorganic conditioner especially dual Fenton oxidation conditioner significantly improved the sludge dewaterability to reach to 51 and 59% CST reduction after using CaSO_4_ and Fenton, respectively. Gypsum acts as skeleton builder that differs than the Fenton or conditioner. Notably, the presence of CaSO_4_ assists to construct a superior porous and inflexible lattice structure of the dewatered *Al-S* cake. Although, in the case of the Fenton conditioning creates crystalloids clumps on the flocs’ external surfaces that might assist in facilitating the water withdrawal from *Al-S*.

Although all tested conditioners improved sludge dewaterability to varying degrees, distinct performance differences were observed depending on their conditioning mechanisms. The ionic liquid (IL) exhibited superior performance in reducing CST and SRF, which can be attributed to its combined electrostatic interactions and structural modification of sludge flocs. In contrast, the anionic surfactant (SDS) showed limited effectiveness, likely due to electrostatic repulsion with negatively charged sludge particles, resulting in weaker charge neutralization and less efficient floc formation.

Polymeric conditioners demonstrated moderate to high performance depending on their charge characteristics. Cationic polymers enhanced flocculation through strong charge neutralization and bridging effects, whereas anionic polymers were comparatively less effective. However, polymer-based conditioning may lead to the formation of loosely structured flocs capable of retaining bound water, thereby limiting complete dewatering efficiency.

Inorganic conditioning using gypsum primarily functioned as a skeleton builder, improving sludge permeability and filtration behavior rather than promoting particle destabilization. While this enhances drainage, its influence on electrostatic interactions remains limited compared to organic conditioners. Similarly, Fenton-based conditioning facilitates partial oxidation of organic matter and extracellular polymeric substances (EPS), promoting water release; however, its effectiveness is highly dependent on operational parameters such as pH and reagent dosage, in addition to the potential formation of secondary sludge.

Notably, the application of a very low dosage of IL achieved highly efficient conditioning performance, significantly reducing chemical consumption. Unlike Fenton-based processes, IL conditioning does not rely on radical oxidation or hydrogen peroxide, thereby minimizing the generation of harmful by-products. The high efficiency at low dosage reflects the strong affinity of IL molecules toward sludge constituents, enabling effective flocculation through electrostatic attraction and surface interactions, even under acidic conditions.

Overall, the superior performance of IL highlights its multifunctional role, combining charge neutralization, floc restructuring, and enhanced water release. In addition, IL conditioning offers operational simplicity and stability compared to conventional methods. Literature reports^33^ further suggest the potential for IL recovery and reuse, supporting its sustainability. Nevertheless, considerations related to cost, scalability, and environmental compatibility particularly with respect to IL composition and potential impacts on microbial systems should be carefully evaluated for practical applications.

The observed improvement in sludge dewaterability can be directly linked to the underlying conditioning mechanisms. The reduction in CST and SRF, together with the shift in zeta potential toward neutrality, indicates effective charge neutralization and enhanced floc aggregation. Additionally, the decrease in turbidity and moisture content suggests improved floc structure and water release, likely due to disruption of extracellular polymeric substances and partial conversion of bound water into free water. Compared to conventional conditioners, the ionic liquid exhibits a combined effect involving electrostatic interactions, floc restructuring, and enhanced permeability, which collectively account for its superior performance.

The improved dewaterability achieved through conditioning has important implications for sludge reuse and valorization. In particular, ionic liquid (IL) conditioning results in a denser sludge structure with reduced moisture content and enhanced solid–liquid separation, which lowers handling and transportation costs. The reduced reliance on oxidative reagents and the absence of significant secondary by-product formation further contribute to improved sludge quality. These characteristics make the conditioned sludge more suitable for potential reuse pathways, such as incorporation into construction materials such as bricks or cementitious composites^23^, use as a precursor for adsorbent materials, or application in soil conditioning, provided that environmental safety and leaching risks are adequately assessed^2^. Therefore, the conditioning process not only enhances dewatering performance but also supports the transition from sludge disposal to resource recovery.

### Comparison of different conditioner for alum sludge

Essentially, alum sludge as a by-product from waterworks discharge must undergo mechanical dewatering to reduce its water content and volume prior to disposal or reuse. In this context, the selection of effective conditioning agents remains a key research focus. Table 2 summarizes the performance of various conventional and emerging conditioners applied for alum sludge treatment. According to the presented data, the proposed green ionic liquid (IL) system achieved a 47% CST reduction, which is comparable to several conventional conditioners, including polymer-based systems (48–51%) and gypsum (about 51%), although slightly lower than Fenton-based conditioning (approximately 59%). However, this comparison highlights important distinctions in terms of process sustainability and chemical requirements. Conventional polymeric and surfactant-based conditioners, despite their high efficiency, are often associated with toxicity, secondary pollution, and higher chemical dosages, whereas Fenton-based systems rely on oxidative reactions that generate additional sludge and require strict pH control. In contrast, the IL-based system operates at an exceptionally low dosage (0.78 mg g⁻^1^-DS) and does not produce secondary chemical sludge or hazardous by-products.

**Table 2:** Comparison of different Alum Sludge conditioner types for improving dewaterability.

Conditioner	Green/Eco-Friendly	Operating pH	CST Reduction (%)	Notes	Reference
Ionic Liquid (IL)	Green	3.2	47.0	Low dosage; effective CST reduction; cost-efficient	Current work
Polymer E0-414	Not Green	7	48.75	Common commercial synthetic polymer	Current work
Polymer LT-25	Not Green	7	51.24	Strong performance in sludge dewatering	Current work
SDS (Surfactant)	Not Green	7	54.85	Anionic surfactant; toxicity concerns	Current work
Fenton Reagent	Not Green	3	59.36	Oxidative treatment, but generates sludge & iron	Current work
Gypsum	Green	~ 7	51.46	Enhances floc strength; widely available	Current work
CTAB	Not Green	-	57	Cationic surfactant	^9^
Chitosan-Magnetite	Green	7	88	Magnetic separation; biodegradable	^50^
Natural Zeolite	Green	6.5	53.5	Effective ion-exchanger; low cost	^51^
MO	Green	6.1	66		^52^
Anionic polyelectrolyt	Not Green	6.1	93	Anionic surfactant; toxicityconcern	^52^
Cu^2+^/ H_2_O_2_	Not Green	6.0	7		^32^

CTAB: Cetyltrimethylammonium Bromide; SDS: Sodium Dodecyl Sulfate; MO: Moringa oleifera.

From a competitiveness perspective, while the IL system may not always achieve the highest absolute CST reduction, it offers a balanced performance combining efficiency with environmental compatibility and resource recovery potential. Notably, the conditioned sludge can be further valorized as an adsorbent material, which is not feasible with most conventional conditioners. Therefore, the proposed approach should be viewed not only as a dewatering enhancement method but also as a multifunctional and sustainable alternative within an integrated treatment framework.

While the improved dewaterability and structural characteristics of the conditioned sludge indicate promising potential for reuse and resource recovery, these applications remain prospective and require further investigation, particularly regarding environmental safety, leaching behavior, and long-term performance^41^.

### Environmental and life-cycle considerations

The environmental implications of the proposed ionic liquid (IL)-based conditioning approach were evaluated from a qualitative life-cycle perspective, considering chemical consumption, process emissions, secondary waste generation, and resource recovery potential. Compared to conventional sludge conditioning methods, the present system demonstrates several environmentally favorable attributes. Initially, the extremely low dosage of the amino acid–based ionic liquid (0.78 mg g⁻^1^ DS) significantly reduces overall chemical input relative to traditional conditioners such as synthetic polymers, surfactants, and Fenton reagents, which are typically applied at substantially higher concentrations. This reduction in chemical demand directly contributes to minimizing upstream environmental burdens associated with chemical production, transport, and handling^1,2^. Additionally, the use of a biodegradable and low-toxicity ionic liquid (choline–glycine) mitigates concerns related to secondary pollution and ecotoxicity, which are commonly associated with conventional polyelectrolytes and surfactants. In contrast to oxidation-based systems (e.g., Fenton processes), the proposed approach does not generate additional chemical sludge or residual oxidants, thereby reducing downstream treatment requirements and disposal impacts.

Further, a key advantage of this system lies in its integration of sludge conditioning with subsequent valorization. The conditioned alum sludge is repurposed as an adsorbent for wastewater treatment, effectively transforming a waste by-product into a value-added material. This strategy aligns with circular economy principles by extending the material life cycle, reducing landfill disposal, and offsetting the need for virgin adsorbent production.

From an energy and process perspective, the conditioning step operates under mild conditions with short contact time, further contributing to reduced operational footprint. Although acidic conditions (pH about 3) are required for optimal performance, the associated environmental impacts are partially mitigated by the low reagent dosage and the potential for process optimization at near-neutral pH. Hence, while a full quantitative life-cycle assessment (LCA) is beyond the scope of this study, the presented results indicate that the proposed IL-based system offers a more sustainable alternative to conventional sludge conditioning methods by combining reduced chemical usage, lower environmental impact, and resource recovery within a unified treatment framework.

### Characteristics and mechanism analysis of *Al-S*

#### The characterization of solid particles

##### XRD pattern of Al-S

**Fig. 5:**
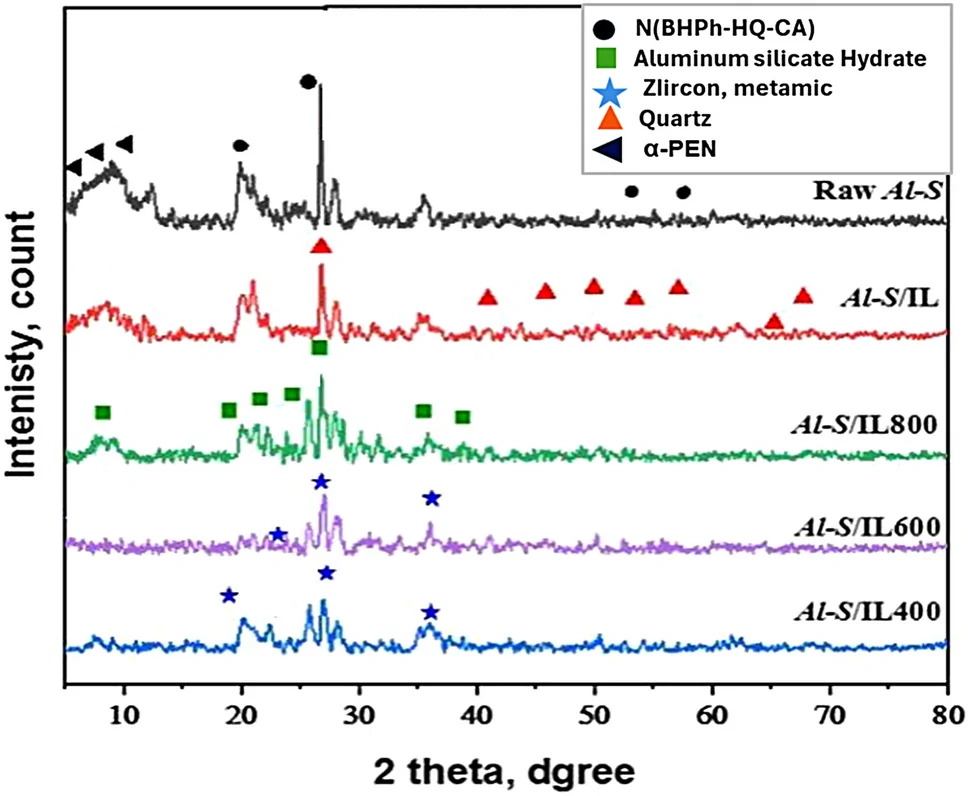
XRD pattern of *Al-S, Al-S* /IL, *Al-S* /IL400, *Al-S* /IL600 and *Al-S* /IL800.

The crystal structures of raw alum sludge (Al-S), ionic liquid-conditioned sludge (Al-S/IL), and the thermally treated products (Al-S/IL400, Al-S/IL600, and Al-S/IL800) were investigated by X-ray diffraction (XRD), and the corresponding diffraction patterns are presented in Fig. 5. The XRD profiles reveal clear structural differences between the raw sludge and the conditioned and calcined samples, demonstrating the influence of ionic liquid treatment and thermal activation on the mineral composition of alum sludge. The diffraction patterns of all samples exhibit distinct crystalline reflections together with a broad amorphous background. Quartz (SiO₂) was identified as the dominant crystalline phase, exhibiting characteristic diffraction peaks corresponding to its hexagonal crystal structure. In addition, calcium aluminosilicate-related phases were detected in all samples, reflecting the inorganic composition of the water treatment residuals^53,54^. The raw alum sludge also exhibited a broad diffraction hump between approximately 15° and 35° (2θ), indicating the presence of amorphous aluminosilicate materials.

Following ionic liquid conditioning, slight changes in the intensity and sharpness of several diffraction peaks were observed, suggesting structural modification of the sludge matrix while preserving its principal mineral phases. Subsequent calcination progressively enhanced the crystallinity of the material, as evidenced by the sharper and more intense diffraction peaks observed for Al-S/IL400, Al-S/IL600, and particularly Al-S/IL800. This behavior is attributed to the thermal transformation of amorphous constituents into more stable crystalline aluminosilicate phases during calcination. The increase in crystallinity at elevated temperatures is consistent with the decomposition of hydrated mineral phases and structural reorganization of the inorganic framework. Hence, the XRD results demonstrate that ionic liquid conditioning followed by thermal treatment effectively modifies the mineral structure of alum sludge by reducing the amorphous fraction and enhancing the crystallinity of the inorganic framework. These structural changes are expected to improve the physicochemical stability of the sludge-derived materials and contribute to their enhanced adsorption performance in wastewater treatment applications^53–55^.

### Al-S SEM Morphological analysis

**Fig. 6:**
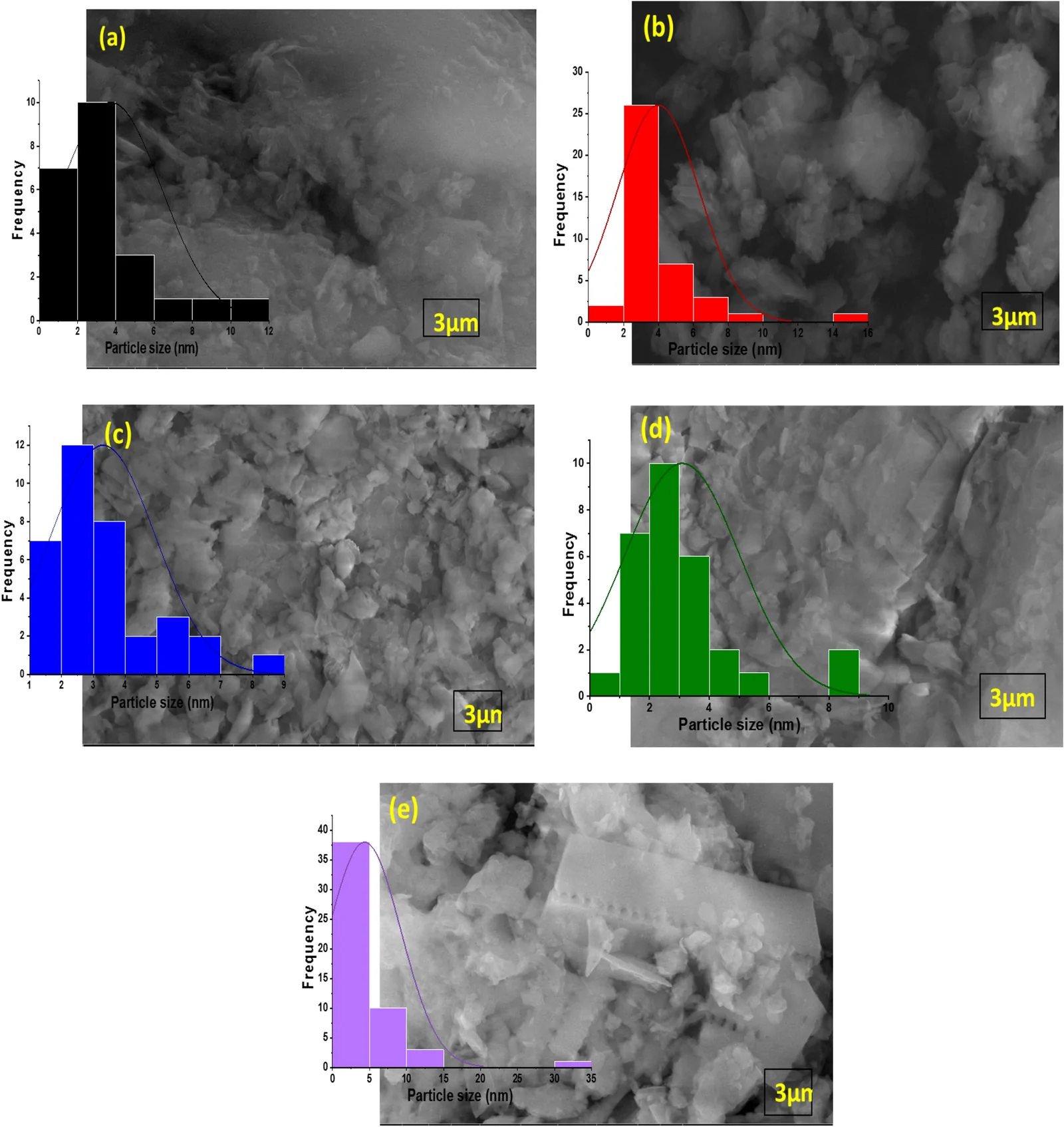
SEM images augmented with the corresponding particle size distribution (inset) of* (****a****) Raw Al-S, (****b****) Al-S* /IL, (**c**) *Al-S* /IL400, (**d**) *Al-S* /IL600, (**e**) *Al-S* /IL800.

The morphological characteristics of raw alum sludge (Al-S), ionic liquid-conditioned sludge (Al-S/IL), and the calcined products (Al-S/IL400, Al-S/IL600, and Al-S/IL800) are presented in Fig. 6 together with their corresponding particle size distributions. The raw Al-S (Fig. 6a) exhibited dense and irregular agglomerates with limited porosity and an average particle size of approximately 3.75 nm, reflecting a compact structure that restricts water release and contributes to poor dewaterability. Following ionic liquid conditioning (Fig. 6b), the sludge structure became more porous and homogeneous, with partial disintegration of compact aggregates and a slight increase in average particle size (3.99 nm). These changes indicate effective floc restructuring and disruption of interparticle interactions, facilitating improved water release during dewatering^31,56^.

Thermal treatment further modified the sludge morphology. Calcination at 400 °C (Fig. 6c) promoted pore development and increased surface roughness through partial decomposition of organic constituents. Increasing the calcination temperature to 600 °C (Fig. 6d) produced a more homogeneous porous structure with improved structural stability, whereas calcination at 800 °C (Fig. 6e) resulted in partial particle sintering and pore collapse, accompanied by an increase in the average particle size to approximately 4.34 nm, indicating structural densification and reduced surface functionality^57,58^. Hence, the SEM observations demonstrate that ionic liquid conditioning and subsequent calcination progressively modify the sludge microstructure by enhancing porosity, reorganizing particle morphology, and improving floc architecture. These structural changes are consistent with the enhanced dewaterability and adsorption performance observed for the treated alum sludge, confirming the effectiveness of the proposed conditioning strategy.

### Energy dispersive X-ray spectroscopy (EDX)

**Fig. 7:**
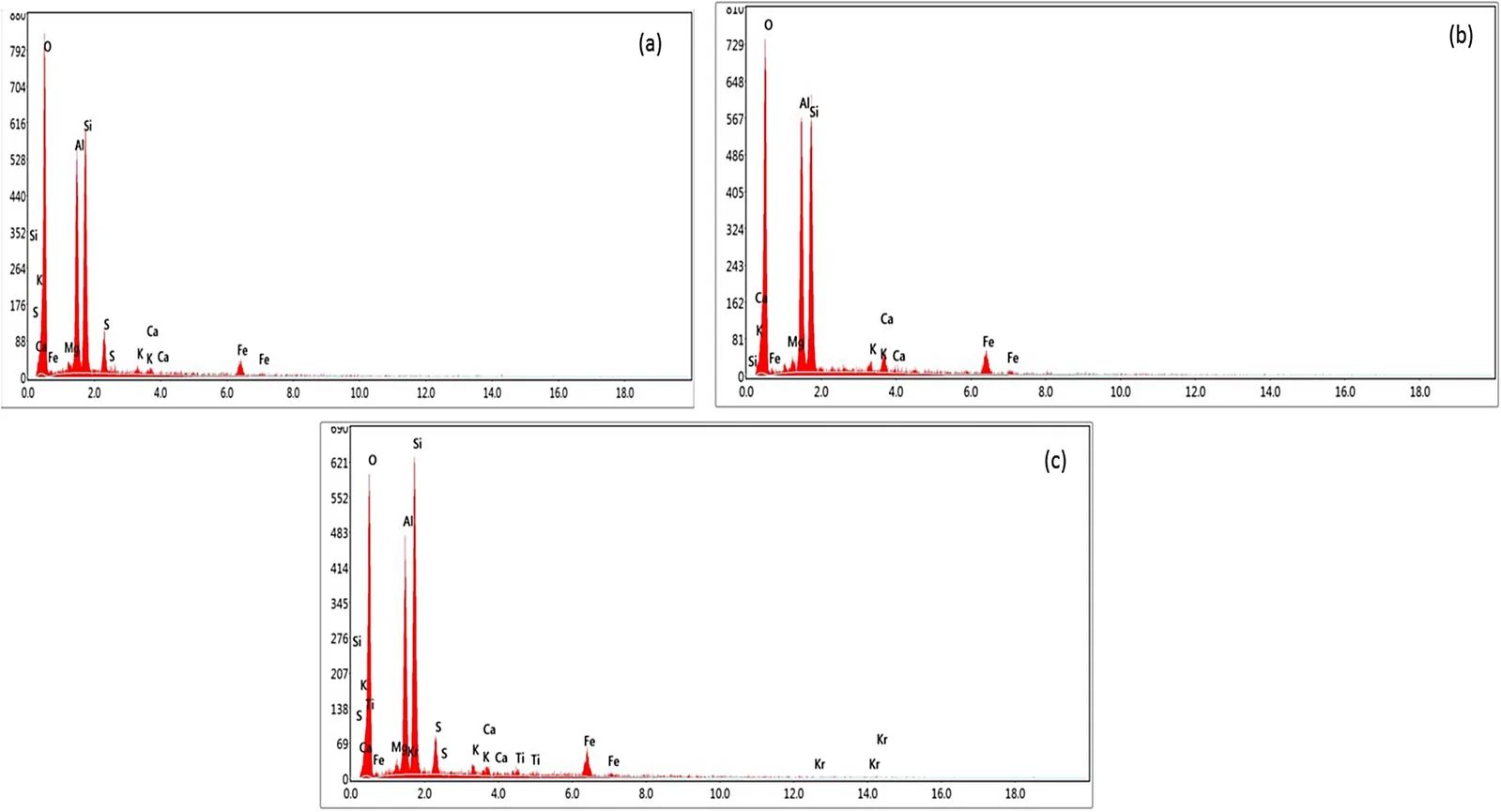
EDX of (**a**) Raw *Al-S* , (**b**) *Al-S* /IL and (**c**) *Al-S* /IL400.

An elemental analysis using Energy Dispersive X-ray Spectroscopy (EDX) was conducted on the three types of alum sludge (raw *Al-S* , *Al-S* /IL and calcined *Al-S* /IL400) to investigate the impact of conditioning with ionic liquids (IL) and subsequent calcination form *Al-S* /IL400 and the data displayed in Fig. 7 a, b and d that corresponding to the data of raw *Al-S* , *Al-S* /IL, and the third and *Al-S* /IL400, respectively. The EDX analysis of the raw alum sludge mainly showed the presence of oxygen (O), aluminum (Al), silicon (Si), along with smaller amounts of sulfur (S), iron (Fe), calcium (Ca), and potassium (K). This composition reflects the typical mineral content of sludge generated from aluminum-based water treatment processes, where aluminum compounds and silicates are the primary constituents. After conditioning with ionic liquids (Fig. 7 b), noticeable changes appeared in the elemental profile. The peaks of Al, Si, and O became more intense, and nitrogen (N) was detected, likely originating from the ionic liquid itself. This suggests that IL treatment altered the surface chemistry of the sludge, potentially dissolving loosely bound impurities or modifying the surface layers. Such behavior aligns with the known chemical activity of ionic liquids towards inorganic materials. For the sludge that was subjected to IL conditioning followed by calcination at 400 °C and the data exhibited in Fig. 7 (c), the EDX spectrum revealed sharper peaks for Al, Si, and O, indicating a cleaner and more stable matrix. The heat treatment likely facilitated the removal of organic residues and volatile components, enhancing the crystallinity of the remaining materials. Minor traces of titanium (Ti) were detected, possibly due to contact with processing equipment. The presence of krypton (Kr) is presumed to be an artifact from the EDX analysis environment rather than a component of the sample.

The combined ionic liquids treatment and calcination processing improved the chemical purity and stability of alum sludge^15^. This enhancement suggests that such treated sludge could have potential applications in industrial sectors such as construction materials or environmental remediation^6,59^.

### FTIR spectral analysis

**Fig. 8:**
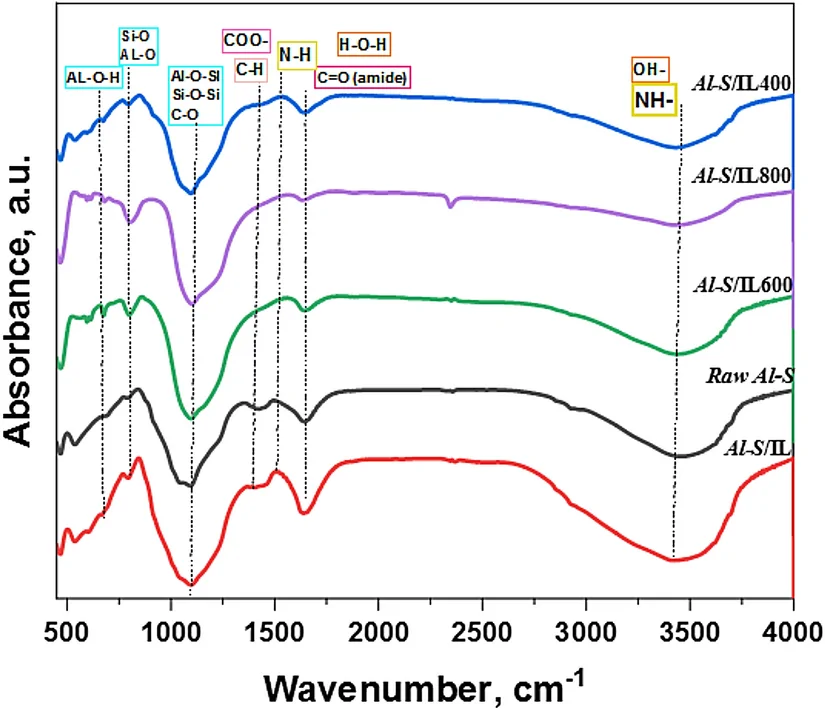
FTIR of* (Raw Al-S , Al-S* /IL, *Al-S* /IL400, (*Al-S* /IL600 and *Al-S* /IL800).

The FTIR spectra in Fig. 8 illustrate the chemical functionalities present in Raw alum sludge *Al-S* , conditioned alum sludge *Al-S* /IL and calcinated form (*Al-S* /IL400, *Al-S* /IL600, *Al-S* /IL800). A broad absorption band observed in the range 3430–3410 cm⁻^1^ is assigned to the stretching vibrations of hydroxyl groups (–OH), typically associated with physically adsorbed water or surface hydroxyls on aluminosilicate structures. Additionally, this region includes N–H stretching from amino acid-derived groups introduced by the ionic liquid^40^. Weak bands appearing around 2923 cm⁻^1^ and 2850 cm⁻^1^ correspond to asymmetric and symmetric stretching of C–H bonds in aliphatic moieties, confirming the incorporation of organic species from the ionic liquid^33^. The distinct peak near 1630 cm⁻^1^ is attributed to C = O stretching of carboxylic or amide groups, and may also reflect H–O–H bending from molecular water. This suggests the presence of amino acid residues or hydrogen-bonded structures in the modified materials^40^. Additional peaks at 1539 cm⁻^1^, 1511 cm⁻^1^, and 1379 cm⁻^1^ are characteristic of COO⁻ asymmetric and symmetric stretching modes, which are commonly found in carboxylate-functionalized materials. These features provided further evidence of successful modification via ionic liquid treatment. A strong band observed around 1096 cm⁻^1^ is assigned to the stretching vibrations of Si–O–Si and Al–O–Si, indicative of silicate and aluminosilicate frameworks inherent to the sludge matrix^56,60^. This band remained consistent across samples, highlighting the structural stability of the inorganic component.

In the lower frequency region (794, 691, 537, and 468 cm⁻^1^), the observed absorptions correspond to bending vibrations of Si–O and Al–O bonds, which are typical for alum-based mineral phases^55^. Following thermal treatment, spectra of the calcined samples (*Al-S* /IL400, *Al-S* /IL800, and *Al-S* /IL800) reveal a gradual reduction in the intensity of organic-related peaks (C–H, N–H, and COO⁻), reflecting the progressive decomposition of the organic modifier. However, the inorganic Si–O and Al–O vibrational modes persist, demonstrating good thermal stability of the core alum sludge matrix.

### BET surface area analysis

BET surface area analysis was performed using the Brunauer–Emmett–Teller (BET) technique to investigate the conditioned alum sludge *Al-S* /IL surface area and the data presented in Table 3 were obtained using nitrogen physisorption at 77 K and the data displayed in Table 3. The resulting isotherm showed a clear linear section between P/P₀ of 0.05–0.30, confirming the model’s validity. Our data revealed a monolayer volume (Vm) of 1.13 cm^3^ (STP)/g and a specific surface area of 4.92 m^2^ g^-1^. Although modest in magnitude, this level of surface area still indicates sufficient availability of adsorption sites for practical applications. The BET constant (C) was 58.9, indicating moderate-to-strong interaction strength between nitrogen molecules and surface sites.

**Table 3:** BET surface area analysis of *Al-S* /IL.

Parameter	Value	Unit
*V*_*m*_	1.13	[cm^3^(STP) g^-1^]
*a*_*s,BET*_	4.9183	[m^2^ g^-1^]
*C*	58.897	
Total pore volume (*p*/*p*_0_ = 0.9900)	0.053168	[cm^3^ g^-1^]
Average pore diameter	43.241	[nm]

Furthermore, calculating from the 4 V/A method provided an average pore diameter of 43.2 nm, classifying the material as mesoporous under the 2–50 nm range defined by IUPAC^61,62^. Such mesoporosity is promising, especially for applications like catalysis supports, dye absorption, filtration systems, and certain types of energy storage, where moderate surface area with accessible pores is a key factor. Although the BET model is widely used for surface area estimations, recent critiques highlight its assumptions and possible 10% systematic uncertainties. Given these limitations, it is essential to treat the BET results as valuable but not absolute complementary analyses (e.g., pore-size distribution) are recommended for a fuller picture^62^.

#### Characterization of supernatant

##### Zeta potential results

**Fig. 9:**
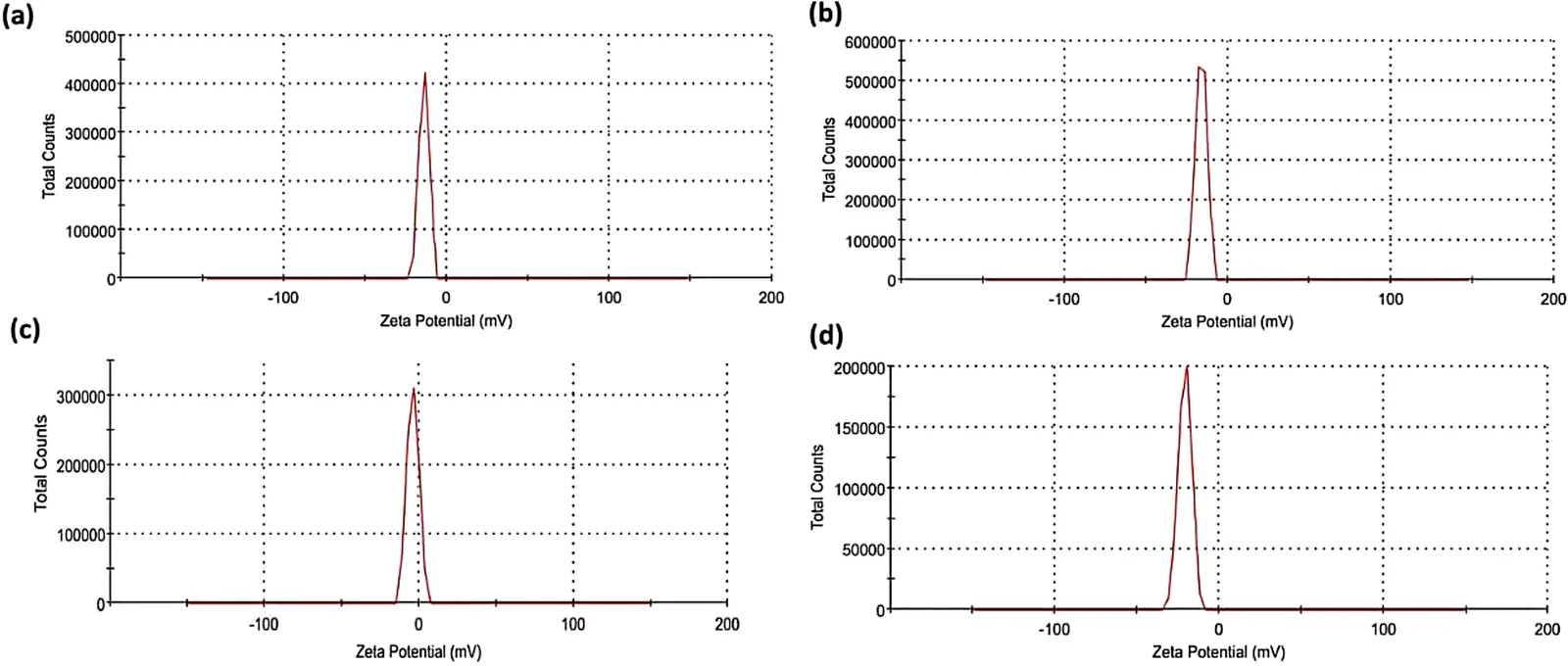
Zeta potential of supernatant of conditioned alum sludge at (**a**) pH 7.5 (raw supernatant), (**b**) pH 8, (**c**) pH 3.2 and (**d**) pH 9.

The zeta potential measurement Fig. 9 were used to evaluate the surface charge of particles in suspension and their colloidal stability. This directly impacts the behavior of sludge particles, especially their ability to aggregate and settle. Generally, when the zeta potential approaches zero, the repulsive forces between particles decrease, leading to enhanced aggregation and improved sludge dewatering^63^. Figure 9 (d) shows the zeta result of the raw alum sludge supernatant.

Figure 9 (a) displays the supernatant raw alum sludge zeta potential data. The data showed that the raw sludge possessed a pH 7.5 shows a slightly negative zeta potential (-14.0 ± 2.99 mV), suggesting limited natural destabilization. Without any chemical conditioning, the sludge maintains moderate stability, resulting in a less efficient coagulation process compared to the optimized acidic condition^64^.

Figure 9 (b) exhibited that the IL addition to *AL-S* changed the pH value from 7.5 to 8.0 results in a change in zeta potential value. In such case the zeta potential of the sludge supernatant reached to -16.3 ± 3.34 mV. A more negative zeta potential indicates stronger particle stability. This means zeta potential is slightly shifted towards the negative side. This slight negativity partially stabilizes the particles, limiting their tendency to aggregate and thus reducing the effectiveness of the dewatering process^65^.

Figure 9 (c) shows the data of conditioned alum sludge at the optimum conditions (acidic pH 3.2), the conditioned alum sludge exhibits a zeta potential close to zero that changed to -4.30 ± 3.78 mV. This neutral charge reduces the electrostatic repulsion between particles, favoring aggregation and promoting effective sludge dewatering. This condition represents the optimum for sludge treatment, as the particles become unstable and tend to coagulate easily^63^.

Figure 9 (d) displays the zeta potential when the conditioned sludge is operated at alkaline pH 8.0 increased to 9.0 after the IL addition. In such case, the zeta potential recorded a value of -20.9 ± 3.97 mV compared to -14 ± 2.99 mV for the raw alum sludge. This condition suppresses particle aggregation significantly, which negatively affects sludge dewatering efficiency^25^.

The results confirm that adjusting the pH of conditioned alum sludge to 3.0 significantly improves particle destabilization by minimizing surface charge. Thus, such mechanism enhances the conditioning performance and thereby improves the dewatering process. In contrast, operating under neutral or alkaline pH conditions, especially at alkali addition, increases the particle stability and reduces coagulation efficiency.

#### Interaction mechanism of [Ch]-[AA] with alum sludge

**Fig. 10:**
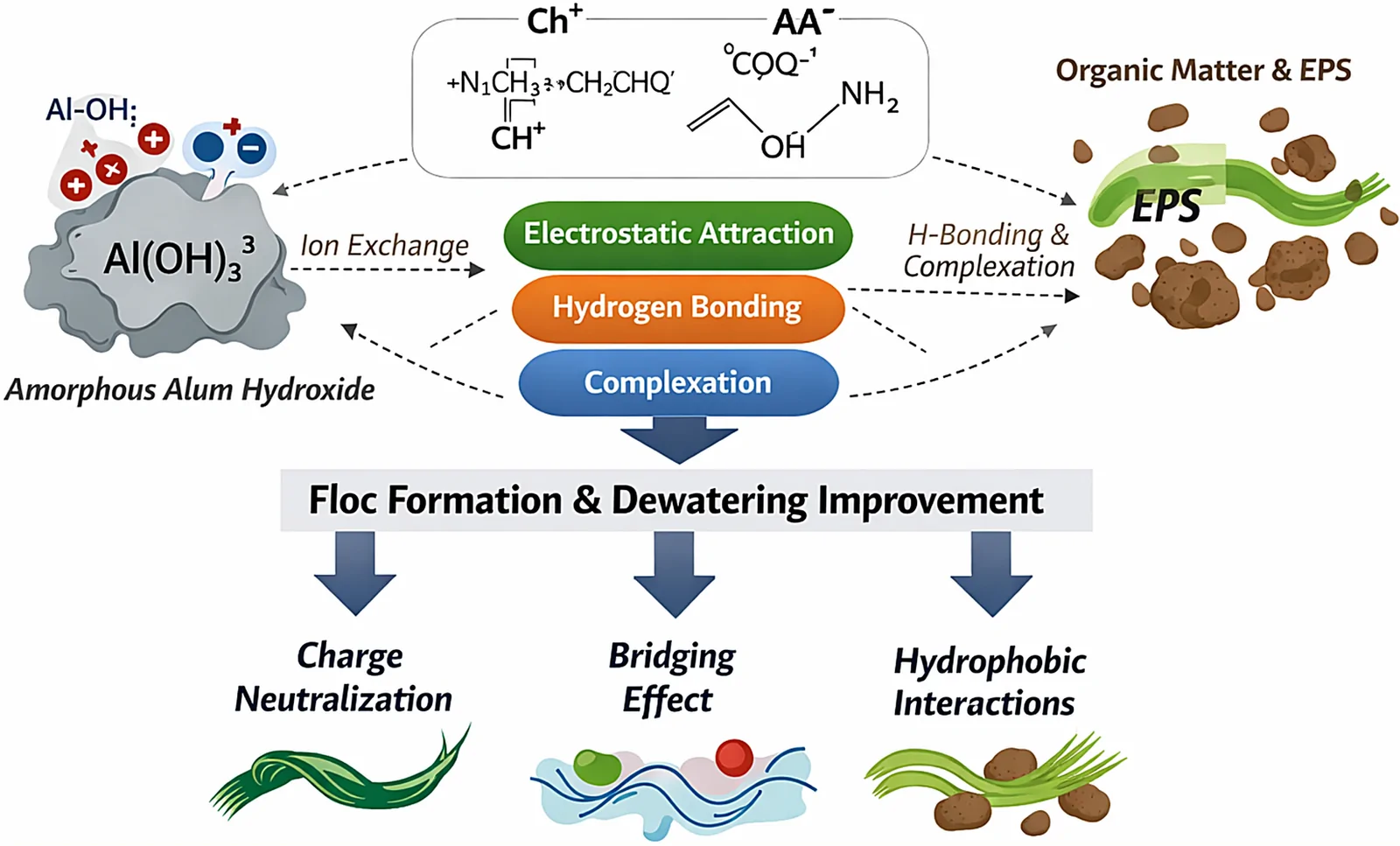
Schematic illustration of electrostatic, hydrogen bonding, and bridging interactions between [Ch]-[AA] ionic liquid and alum sludge component.

To better interpret the observed improvement in sludge dewaterability, the interaction mechanisms between the choline–amino acid ionic liquid ([Ch]-[AA]) and alum sludge components are schematically illustrated in Fig. 10. As shown in Fig. 10, alum sludge is predominantly composed of amorphous aluminum hydroxides (Al(OH)₃) with surface hydroxyl groups exhibiting pH-dependent charge characteristics. The [Ch]-[AA] ionic liquid, consisting of positively charged choline cations ([Ch]⁺) and amino acid-derived anions ([AA]⁻), interacts with these surfaces through electrostatic attraction and ion exchange, leading to partial charge neutralization. Consistent with the observed zeta potential shift, the degree of destabilization appears to be partial rather than complete, suggesting that charge neutralization alone is not the dominant mechanism. In addition, the amino acid moieties containing functional groups such as –COO⁻ and –NH₂, facilitate hydrogen bonding and complexation with aluminum species, while also interacting with organic matter and extracellular polymeric substances (EPS) within the sludge matrix. These combined interactions promote particle aggregation and floc strengthening through bridging effects, resulting in the formation of larger and more compact flocs.

Consequently, enhanced sludge dewaterability was achieved due to improved solid–liquid separation and reduced bound water content. Furthermore, the amphiphilic nature of [Ch]-[AA] may contribute to the disruption of bound water and the reorganization of EPS structure, further improving the overall conditioning performance.

### Dye containing wastewater adsorption

#### Synozol KHL Red and Blue treatment

##### Adsorption time-profile

**Fig. 11:**
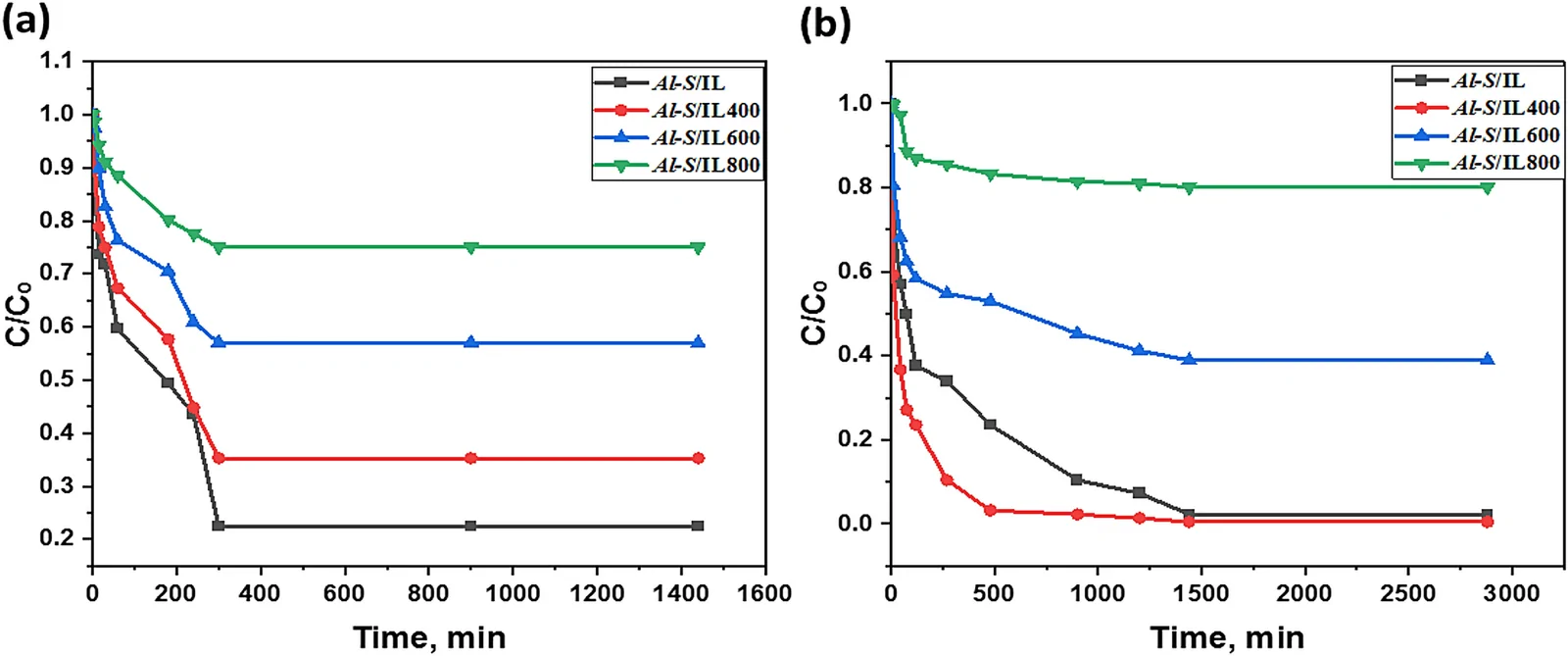
Adsorption time-profile using various adsorbents for (**a**) Synozol KHL Red and (**b**) Synozol KHL Blue removals (adsorbent dose: 0.5 g L^-1^; dye concentration 30 ppm and pH 7.0 at room temperature).

Initially, in order to understand the adsorption matrix and optimize process dynamics, it was essential to investigate the influence of contact time on dye removal performance. In this regard, the time profile of two synthetic dyes namely Synozol KHL Red and Synozol KHL Blue, were studied at room temperature using IL-conditioned alum sludge (*Al-S* /IL) and its thermally modified systems calcined at the appropriate temperature 400 °C, 600 °C, and 800 °C and shown in Fig. 11.

The isothermal time profiles are illustrated in Fig. 11 simultaneously comparing the adsorption performance across the four adsorbents for both dyes. The data revealed that the majority of the dye uptake occurred during the initial phase, particularly within the first 5 h for Synozol KHL Red and the isotherm time was identified at 24 h for the Synozol KHL Blue dye. However, as time progressed, plateau were attained. This could indicate that the equilibrium time is attained. The considerable difference in equilibrium contact time between the two dyes can be explained by differences in their molecular structure, size, and interaction with the adsorbent surface. Dyes with larger molecular size and more complex structure tend to diffuse more slowly through the pores of the adsorbent, which prolongs the time needed to reach saturation. In contrast, dyes with smaller molecular size and simpler structure can penetrate the pore network more easily, leading to faster equilibrium. Such differences in the isotherm time for both dyes might also be related to the electrostatic interactions also played a critical role. furthermore, when the dye that exhibits greater electrostatic attraction to introduced adsorbent’s surface might occupy active sites more rapidly than one with weaker surface affinity under the same pH and ionic strength conditions^66^.

It is noteworthy to mention that among the introduced adsorbents *Al-S* /IL and *Al-S* /IL400 materials exhibited superior adsorption performance in comparison to the thermally treated materials at high temperature signified as *Al-S* /IL600 and *Al-S* /IL800. This could be attributed to the enhanced behavior is likely ascribed to the retention of the surface with functional groups and structural porosity. Such characteristics are essential for efficient dye uptake. In contrast, the reduction in efficiency observed with *Al-S* /IL 600 and 800 is probably due to pore collapse and thermal degradation of surface sites. Such findings confirm that contact time plays a critical role in the rapid dye adsorption and that the early stage governs the majority of dye removal due to abundant surface activity. This phenomenon has been previously reported by other researchers^67^.

### Effect of initial dye loading

**Fig. 12:**
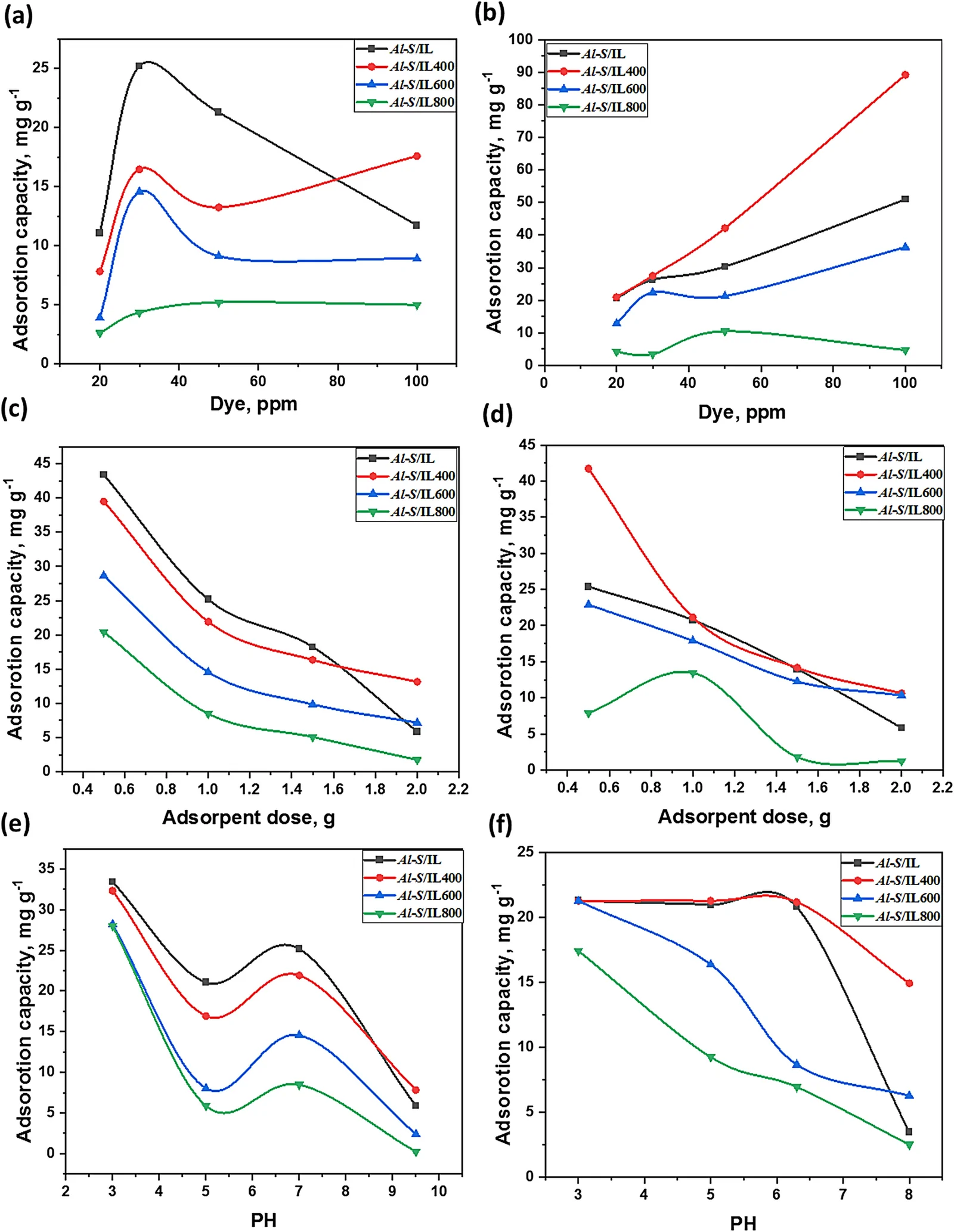

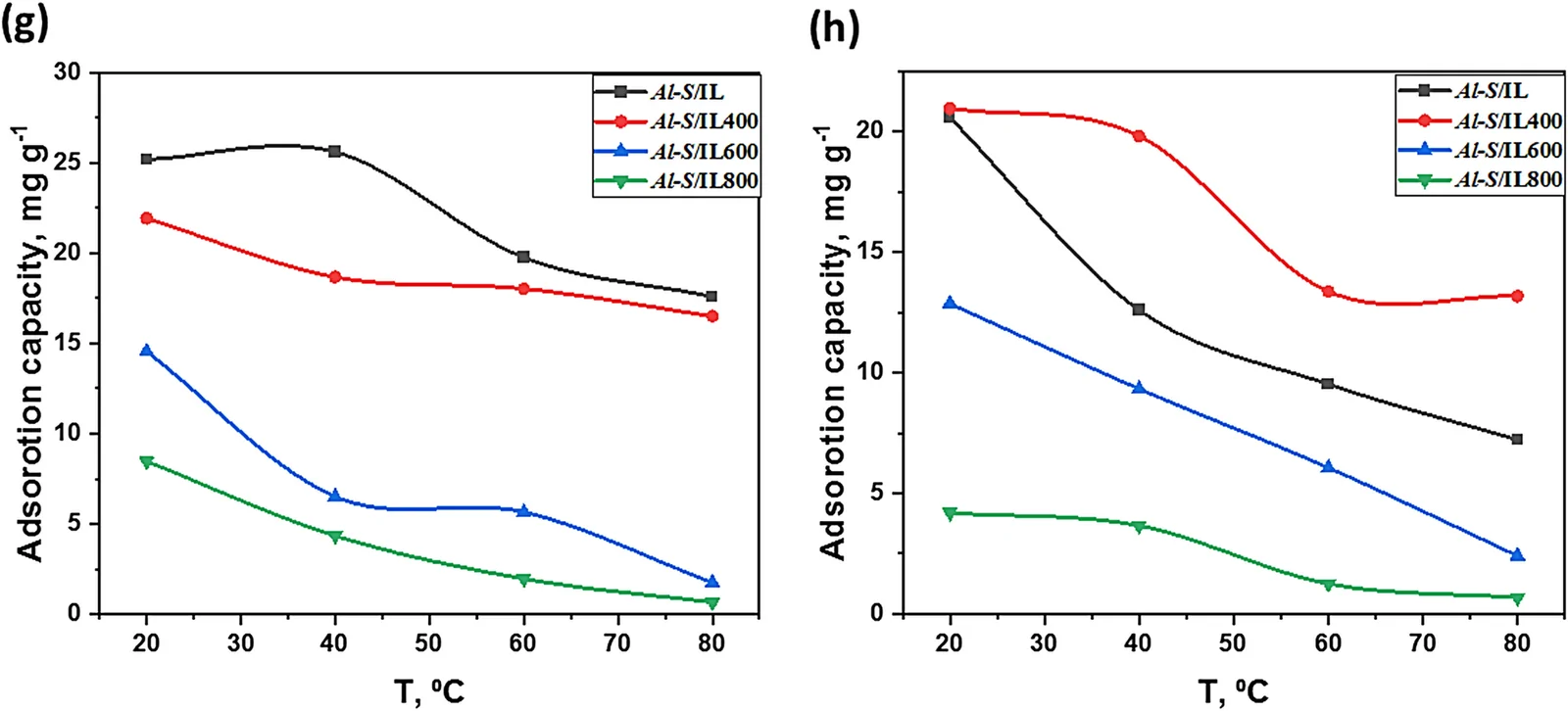
Effect of adsorption parameters using various adsorbents for Synozol KHL Red and Synozol KHL Blue removals (**a**) Synozol KHL Red dye concentration (adsorbent dose: 1 g L^-1^;and pH 7.0), (**b**) Synozol KHL Blue dye concentration (adsorbent dose: 1 g L^-1^ and pH 6.3), (**c**) adsorbent dose (Synozol KHL Red dye concentration 30 ppm and pH 7.0), (**d**) adsorbent dose (Synozol KHL Blue concentration 30 ppm and pH 6.3), (**e**) pH (Synozol KHL Red dye concentration 30 ppm and adsorbent dose 1 g L^-1^) and (**f**) pH (Synozol KHL Blue dye concentration 30 ppm and adsorbent dose 1 g L^-1^), (**g**) temperature Synozol KHL Red dye concentration 30 ppm and adsorbent dose 1 g L^-1^ and pH 7.0), (**g**) temperature (Synozol KHL BLUE dye concentration 30 ppm and adsorbent dose 1 g L^-1^ and Ph.

For real-scale applications, it is essential to investigate the influence of pollutant loading on the sorption capacity and performance. In this context, the influence of initial two studied dye content loading was examined within the range of 10 to 100 ppm using the developed adsorbents: *Al-S* /IL, *Al-S* /IL400, *Al-S* /IL600, and *Al-S* /IL800. The experimental results displayed in Fig. 12 (a) and (b) clearly illustrate that increasing the initial concentration of Synozol KHL red and Synozol KHL Blue dyes, increased the adsorption capacity. The results depicted in the Fig. 12 (a and b) clearly showed an enhancement in the adsorption capacity (qₑ) for both Synozol KHL red and Synozol KHL Blue dyes. Generally, all tested adsorbents demonstrated a consistent increase in adsorption capacity as dye concentration increased. This trend was more pronounced for Synozol Blue, where *Al-S* /IL that achieved a maximum uptake of approximately 90.1 mg g^-1^ at 100 ppm, followed by *Al-S* /IL400. This could be attributed to the increased interaction between dye molecules and the adsorbent surface at higher concentrations. The elevated dye loading enhances the mass transfer rate between the dye solute and the active surface sites, leading to improved sorption performance^18^. However, a corresponding decline in removal efficiency was also observed with increasing dye concentration. This reduction can be explained by the fixed adsorbent mass used in the system, which becomes insufficient to accommodate the excess number of dye molecules in solution. Once the active sites are saturated, additional dye molecules remain unadsorbed in the medium, resulting in a decline in removal percentage. This behavior was particularly evident for the thermally treated samples, especially *Al-S* /IL800, which exhibited limited uptake due to pore collapse and loss of surface activity.

**Fig. 13:**
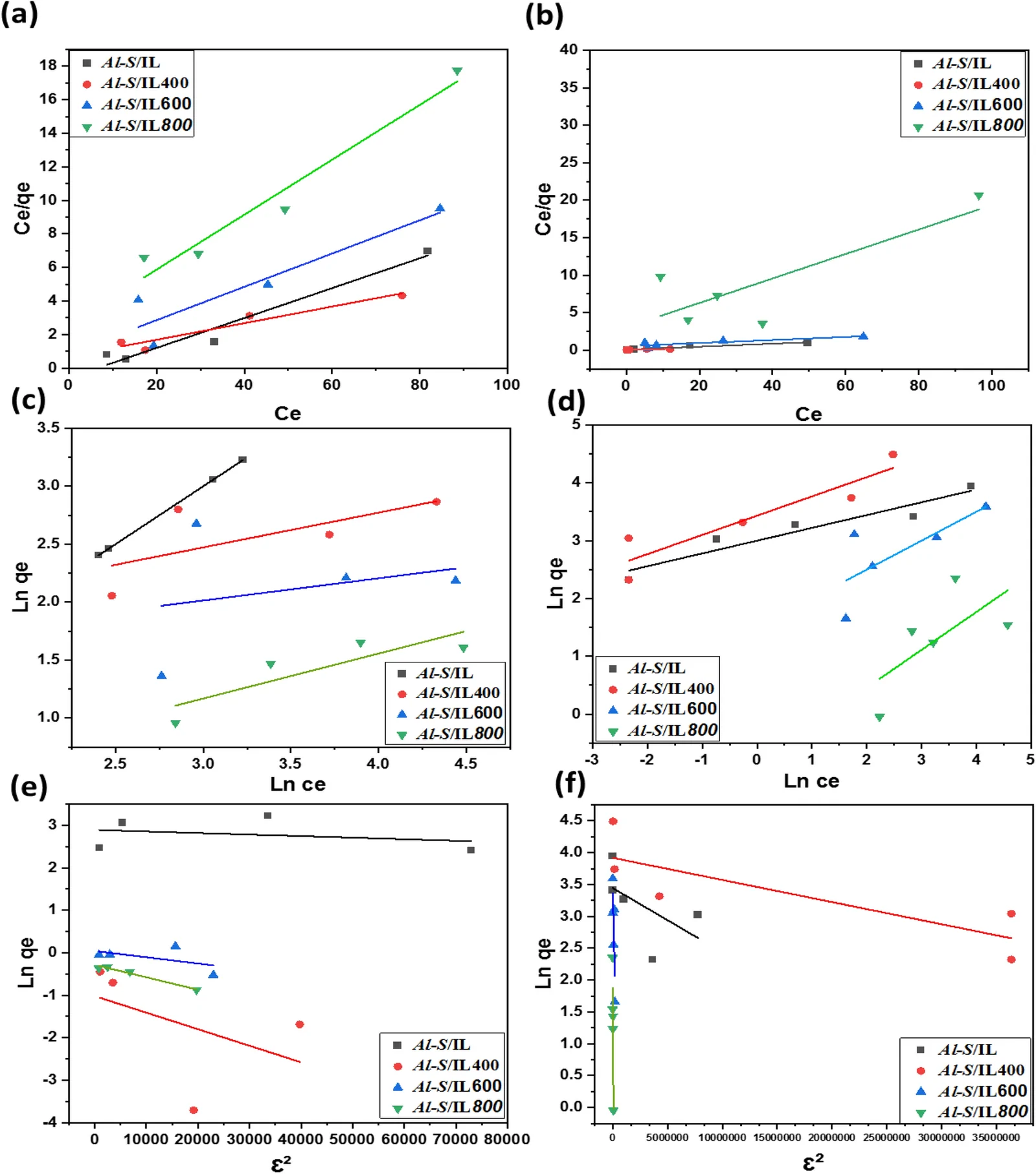

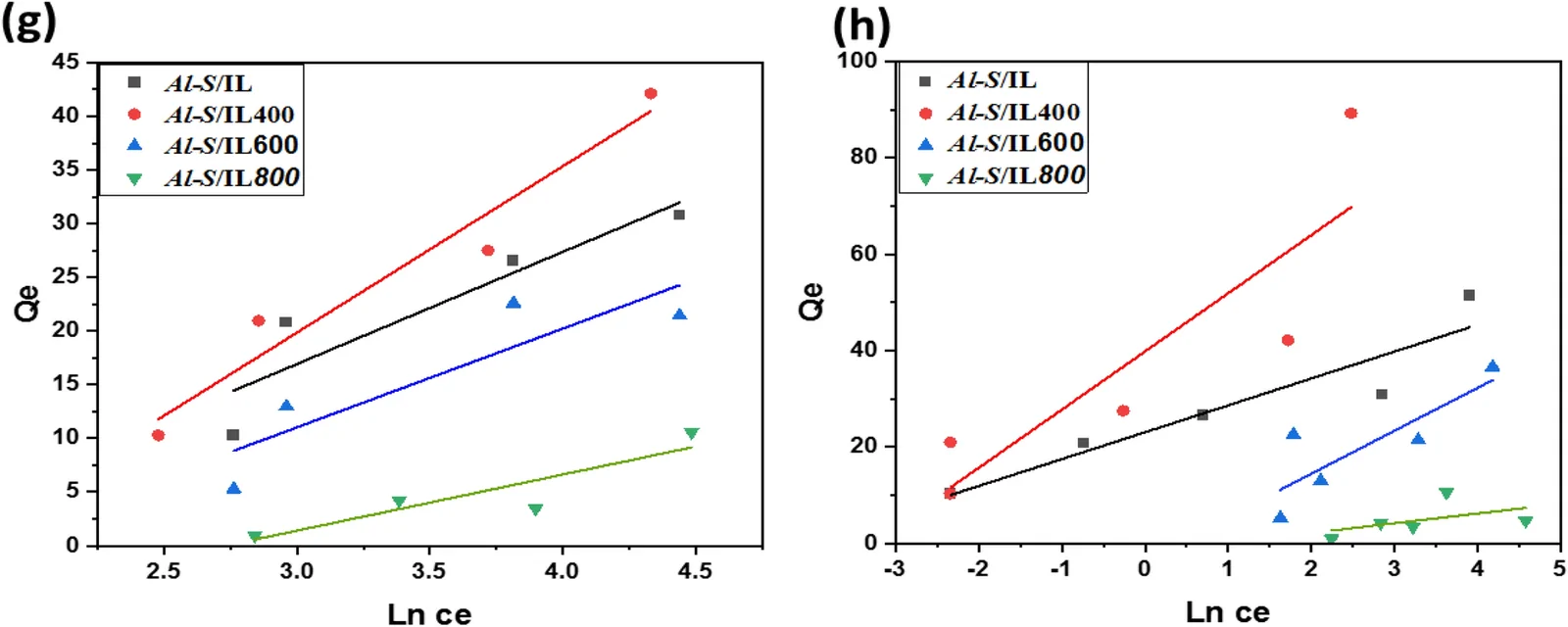
Linearized adsorption isotherm for varied adsorbent according to (**a**) Langmuir Synozol red and (**b**) Blue K-HL, (**c**) Freundlich Synozol red and (**d**) Blue K-HL, (**e**) D-R Synozol red and (**f**) Blue K-HL, (**g**) Temkin Synozol red and (**h**) Blue K-HL.

The adsorption capacities in Fig. 13 a that are corresponding to Synozol KHL Red dye uptake demonstrated a noticeable trend across increasing dye concentrations (20–100 ppm), with a peak performance at 30 ppm for all samples. This behavior is consistent with the general adsorption mechanism where, at lower dye concentrations, sufficient active adsorption sites are available on the adsorbent surface, allowing efficient dye uptake. However, as the dye concentration increases, a saturation of active sites occurs, leading to a reduction in removal efficiency due to site exhaustion or surface coverage limitations^17,68^.

It is essential to mention that, for the IL-conditioned alum sludge thermally treated at 400 °C (*Al-S* /IL400), an initial drop in adsorption capacity was observed at 40 ppm, followed by an increase again at 100 ppm. This non-linear behavior may be attributed to surface structural rearrangements upon moderate thermal treatment. At intermediate concentrations, dye aggregation or steric hindrance could limit accessibility to active sites. But, at high concentration (100 ppm), the increased concentration gradient may overcome these resistances; enhancing the mass transfer rate and facilitating dye diffusion into micropores or newly formed mesopores due to thermal decomposition^69^. Meanwhile, the *Al-S* /IL800 sample exhibited consistently low adsorption performance, which can be explained by significant pore collapse or loss of functional groups at high pyrolysis temperatures, resulting in a reduction in surface area and available adsorption sites^70^.

These findings are in agreement with prior reports indicating that high initial dye concentrations can improve adsorption capacity but may also compromise removal efficiency due to saturation phenomena^67,71^.

### Effect of adsorbent dose

The amount of dye removed from solution is greatly influenced by the applied dosage of the adsorbent. In this investigation, different dosages of the prepared materials were examined within the range of 0.5 to 2.0 g L^-1^ for both dyes Synozol KHL Red and Synozol KHL Blue, while all other operating parameters were kept constant.

As illustrated in Fig. 12 (c) and (d), increasing the adsorbent dosage generally led to a reduction in the adsorption capacity (qₑ, mg g^-1^) for both dyes Synozol KHL Red and Synozol KHL Blue, respectively, across all tested materials. This inverse relationship can be attributed to the increased aggregation of adsorbent particles at higher dosages, which limits the available surface area and may block active adsorption sites. Furthermore, with fixed dye concentrations, the number of dye molecules becomes insufficient to saturate the larger number of active sites, resulting in underutilization of the added adsorbent. The highest adsorption capacities were recorded for *AL-S* /IL400 at the lowest tested dosage (0.5 g L^-1^), with approximately 41.7 mg g^-1^ for Synozol Blue and 43.6 mg g^-1^ for Synozol Red using *AL-S* /IL. This was followed by *AL-S* /IL400, while the thermally treated *AL-S /*IL 800 showed the lowest performance, likely due to structural damage and loss of surface functionality induced by calcination. The results highlight the importance of optimizing adsorbent dosage based on material properties to avoid excessive aggregation or waste of material^72^.

These findings are consistent with previous reports indicating that increasing adsorbent mass beyond an optimal limit does not proportionally enhance dye removal, and may even reduce adsorption efficiency due to overlapping or crowding of particles^27,73,74^.

### Effect of pH on the dyes adsorption

Aqueous solution pH is one of the most influential parameters affecting adsorption behavior due to its role in modifying the surface chemistry of both the adsorbent and the dye molecules. In this regard, variations in pH can influence the degree of ionization, electrostatic interactions, and chemical dissociation of dye molecules, while simultaneously altering the charge distribution and active functional groups on the adsorbent surface^75^.

In the present study, the adsorption behavior of Synozol KHL Blue and Synozol KHL Red was assessed over a pH range from 2.0 to 10.0 using different AL-S /IL-based adsorbents. As displayed in Fig. 12 (e and f), the adsorption capacity for both dyes Synozol KHL Red and Synozol KHL Blue dyes, respectively, exhibited a sharp decline with increasing pH. The adsorption behavior of Synozol KHL Red and Synozol KHL Blue dyes on alum sludge conditioned with ionic liquid demonstrates distinct trends across the pH spectrum, highlighting the influence of dye structure and surface interactions on removal efficiency. At pH 3, Synozol KHL Red exhibits significantly higher adsorption compared to KHL Blue due to its richer anionic nature, particularly the presence of multiple sulfonate (-SO₃⁻) groups that strongly interact with the protonated surface of the adsorbent. The strong electrostatic attraction under acidic conditions enhances the uptake of KHL Red, while KHL Blue, which carries fewer negative charges and possibly a more stable configuration, shows only moderate adsorption^38,76^.

As the pH increases to 5, the surface of the adsorbent becomes less positively charged, and electrostatic attraction decreases for both dyes. However, the drop in adsorption is more noticeable for KHL Red, given its reliance on electrostatic interactions. In contrast, KHL Blue exhibits a relatively stable performance, suggesting other interaction mechanisms such as hydrogen bonding or hydrophobic interactions might be more involved in its adsorption behavior^77^. At neutral pH (pH 7), Synozol KHL Red regains part of its adsorption efficiency, potentially due to non-electrostatic interactions such as π–π stacking and hydrogen bonding supported by the aromatic rings in the dye structure and functional groups on the IL-modified sludge. KHL Blue, however, continues to exhibit a flatter adsorption trend with minor changes, further emphasizing its lesser sensitivity to pH variation^78^. These data are supported by zeta potential results (Fig. 9).

At alkaline pH (pH 9), both dyes show reduced adsorption, with Synozol KHL Red experiencing a more pronounced decline. This is attributed to the increasing negative charge on both the dye molecules and the adsorbent surface, which leads to electrostatic repulsion. Moreover, competition from hydroxide ions for adsorption sites may also contribute to the lower dye uptake. While KHL Blue is also affected by these conditions, the reduction in its performance is not as severe, possibly due to differences in dye charge distribution and molecular conformation^79,80^. Overall, Synozol KHL Red demonstrates a more pH-responsive adsorption profile compared to Synozol KHL Blue. The high charge density and extended conjugation in KHL Red make it more dependent on the electrostatic and π–π interactions, while KHL Blue maintains more consistent, though generally lower, adsorption across varying pH levels. These differences are crucial for optimizing adsorption processes depending on the target dye and environmental conditions.

From an application standpoint, the feasibility of operating under acidic conditions (optimal pH 3.0) warrants careful consideration for large-scale implementation. In the present system, however, the required pH adjustment is partially mitigated by the intrinsic buffering capacity of alum sludge, which can reduce the overall acid demand relative to conventional homogeneous treatment processes. Furthermore, the experimental findings demonstrate that satisfactory dewatering performance can still be achieved under near-neutral conditions (pH 6–7), albeit with a moderate decline in efficiency, thereby providing operational flexibility. From an economic perspective, the additional acid requirement may be partially offset by the exceptionally low dosage of ionic liquid (0.0125 mL L⁻^1^), which substantially reduces total chemical consumption compared to conventional conditioning agents. Although operation at low pH may introduce corrosion-related concerns, such conditions are commonly encountered in established processes including Fenton-based treatment, where appropriate material selection and process design effectively mitigate associated risks. Overall, while acidic conditions favor optimal performance, the proposed system remains adaptable and technically feasible for practical wastewater treatment applications.

### Temperature effect

Since textile dye effluents are generated and discharged over a wide range of temperatures and almost high values^61^, studying the influence of thermal conditions on the adsorption system is crucial. Temperature significantly affects adsorption mechanisms and is a determining factor in practical wastewater treatment applications. To investigate the thermal behavior of the proposed alum sludge-based adsorbents, experiments were conducted in the range of 25–80 °C.

As shown inFig. 12 (g and h), the adsorption capacity of Synozol KHL Red dye increased with rising temperature across all adsorbents, suggesting that the adsorption process is endothermic in nature. Such improvement can be attributed to enhanced molecular motion and an increase in the number of accessible active sites as a result of slight pore expansion. In contrast, for Synozol KHL Blue, the adsorption capacity decreased with temperature, particularly for *AL-S* /IL and *AL-S* /IL400, indicating an exothermic nature of adsorption. This trend implies a weakening of the interaction between dye molecules and adsorbent surfaces as thermal energy increases.

*AL-S* /IL exhibited the highest uptake of Synozol Red at 60 °C, while the lowest performance was observed for *AL-S* /IL 800 due to thermal degradation of active functionalities. The variation in adsorption with temperature could be linked to differences in dye structure and adsorbent surface chemistry. These findings are in line with previous reports that demonstrated endothermic and exothermic patterns for structurally distinct dyes depending on the adsorbent nature and thermal stability^18,69^.

#### Isotherm models

The experimental results were analyzed using the linearized forms of four well-known adsorption isotherm models namely, Langmuir (Eq. 1), Freundlich(Eq. 2), Temkin (Eq. 3) and Dubinin–Radushkevich (D–R) (Eq. 4), to explore the adsorption mechanisms of Synozol KHL Red and Synozol KHL Blue dyes onto *AL-S* /IL and its calcined forms (*AL-S* /IL400, *AL-S* /IL 600 and *AS.IL*800) according to the following equations:

1 $$\frac{{C}_{e}}{{q}_{e}}=\frac{1}{{K}_{L}}+\frac{{a}_{L}}{{K}_{L}}{C}_{e}$$

where:* a*_*L*_*, K*_*L*_ : Langmuir parameters; monolayer adsorption capacity: ${Q}_{o}=\frac{{K}_{L}}{{a}_{L}}$; equilibrium parameter: ${R}_{L}=\frac{1}{{1+K}_{L }{C}_{o}}$ R_L_ that signifies nature of adsorption.

2 $$\mathrm{ln}\left({q}_{e}\right)=ln{K}_{F}+\frac{1}{n}ln{C}_{e}$$

where:* K*_*F*_: Freundlich constant; *n*: heterogeneity constant that illustrates the adsorption isotherm.

3 $${q}_{e}=B lnA+B ln{C}_{e}$$

where: B: related to the heat of adsorption ($B=\frac{\mathrm{R}\mathrm{T}}{\mathrm{b}}$), T: absolute temperature, R is a gas constant;

4 $${lnq}_{e}=ln{q}_{m}-{K}_{DR}{\varepsilon }^{2}$$

where: *q*_*m*_: the monolayer saturation capacity, *K*_*DR*_: D-R isotherm adsorption energy constant, ${\varepsilon }^{2}=RTln(1+\frac{1}{{C}_{e}})$ and *E*: mean free energy of sorption ($E=\frac{1}{\sqrt{2{K}_{DR}}}$) suggests sorption type.

The experimental data were fitted to the selected models and the isotherm constants and correlation coefficients (*R*^*2*^) derived from these models were summarized in Fig. 13, and Table 4. According to the correlation coefficients (*R*^*2*^) of Langmuir isotherm the data verified a high well-fitting of the experimental results with the Langmuir isotherm model for both dyes that signify a high correlation coefficient values reached 0.96 and 0.95 for Synozol KHL Red and Blue, respectively that adsorbed on *AL-S* /IL ranged. This means Freundlich, Temkin and D-R isotherm are less suitabl than the Langmuir isotherm. Such data indicate that the adsorption is following monolayer adsorption on homogeneous surfaces, particularly when *Al-S* /IL material is applied. Also, such data confirmed by regression coefficient is additionally verified for Synozol KHL Red Root Mean Square Error (RMSE) that is commonly used to verify the experimental data. RMSE value for Synozol KHL Red is 0.6 and the corresponding value for Synozol KHL Blue is 0.1 for *Al.S*/IL adsorbent material confirming that the model is following Langmuir isotherm model since the minimal values of the RSME mean the model is accepted^67,76^. Also, the adsorption is favorable when the n value ranges from 1 < n < 10, as displayed from Table 4, n values are greater than unity which signifies the sorption nature is favorable. Interestingly, this aligns with the general criteria where n > 1 verifies a spontaneous and advantageous sorption uptake. The small values of the mean free energy of adsorption (*E*) not more than 8 kJ/mol, assuming the dye adsorption on *AL-S* /IL is a physisorption type; since the forces involved in physisorption are weak Van der Waals forces^39^. The low values of *B*, the heat of adsorption, confirm the physical adsorption process. However, according to the data displayed in Table 4 unusually high values, particularly for Synozol KHL Blue (e.g., *Al-S* /IL: E = 3792.9 kJ/mol) indicate possible deviations or strong interactions that may hint toward chemisorption. Nevertheless, for most realistic values (e.g., *Al-S* /IL Blue at 128.8 kJ/mol), the adsorption may involve both physisorption and chemisorption, governed by van der Waals forces and surface bonding mechanisms^79,81^.

**Table 4:** Isotherm parameters for *Synozol KHL Red and Blue* dyes’ adsorption on *Al-S* /IL and its calcined forms (*Al-S* /IL400,* Al-S* /IL600 and *Al-S* /IL800).

Isotherm parameter	Al-S /IL	Al-S /IL 400	Al-S /IL 600	Al-S /IL 800
**Synozol KHL Red**				
**Langmuir**				
q_m_	1.711	20.18	10.08	6.109
R_L_	0.3259	0.0516	0.1138	0.063
R^2^	0.96	0.93	0.90	0.96
**Freundlich**				
K_F_	1	4.816	4.214	1.002
n	1	3.331	5.212	2.573
R^2^	1	0.4661	0.075	0.075
**D–R**				
KDR	17.99	0.3602	1.04054	0.750152
q_m_ (mol g^-1^)	17.99	0.3602	1.04054	.75015
E (KJ mol^-1^)	370.1	113.1953	184.0127	130.8206
R^2^	0.085	0.22102	0.29333	0.98
**Temkin**				
B (J mol^-1^)	10.45	15.51	9.206	5.23
A (L g^-1^)	3.977	5.583	6.062	15.37
R^2^	0.85	0.95	0.78	0.81
**Synozol KHL Blue**				
**Langmuir**				
q_m_	50.83	86.81	49.55401	6.128578
k_l_	0.341	0.6037	0.037798	0.053842
R^2^	0.95	0.81	0.78	0.66
Frendlish				
K_F_	20.06	30.93	4.477	2.352
n	4.521	3.004	2.001	1.5262
R^2^	0.91	0.85	0.558	0.4410
**D–R**				
q_m_ (mol g^-1^)	31.3	50.32	28.94	6.645
K_D-R_ (mol^2^ J^-2^)	1.01E-07	3.48E-08	6.47E-06	3.01E-05
E (KJ mol^-1^)	2226	3792	277.9	128.9
R^2^	0.32	0.68	0.59	0.79
**Temkin**				
B (J mol^-1^)	3.87	6.450	5.675	6.0712
A (L g^-1^)	268.0	111.8	1.71	8.93
R^2^	0.93	0.88	0.28	0.76

Freundlich and Temkin models further suggest partial surface heterogeneity and variable energy interactions. The D–R energy values indicate that the adsorption mechanism ranges from physical to chemical, depending on dye type and sludge treatment. These findings are consistent with adsorption kinetics^67^, and equilibrium studies in dye removal literature. Thus, the results confirm the Langmuir model is the best-fitting model for both Synozol dyes, particularly with *Al.S*/IL, indicating monolayer adsorption on energetically uniform surfaces.

#### Adsorption thermodynamic

The intrinsic energy changes associated with the adsorption process are thoroughly explained by the thermodynamic parameters. The thermodynamic investigation was conducted to examine the thermodynamic characteristics and nature of the adsorption system. To ascertain the nature of the adsorption process onto the prepared adsorbents, changes in free energy of adsorption (ΔG), enthalpy change (ΔH), and entropy (ΔS) were calculated. The thermodynamic parameters were investigated using the following equations from the Langmuir isotherm parameters, where K_L_ is the equilibrium constant, qe is the amount of dye adsorbed on the adsorbent per liter of solution at equilibrium (mg L^-1^) and Ce is the equilibrium concentration in solution (mg L^-1^).

5 $$ InK_{L} = \frac{\Delta S}{R} - \frac{\Delta H}{{RT}} $$

6 $$ \Delta G = - RT\,InK_{L} $$

where R is the universal gas constant (8.314 J (K mol^-1^))^-1^ and T is the absolute temperature (K). ΔG (kJ mol^-1^), ΔH (kJ mol^-1^) and ΔS J (K mol^-1^)^-1^ indicate changes of Gibbs free energy, enthalpy and entropy, respectively. The plot of ln KL versus 1/T resulted in a straight line (plot not shown), and the values of ΔH and ΔS were calculated from the slopes and intercepts of such plot along with ΔG values (as shown in the Table 5).

**Table 5:** Thermodynamic parameter for dye adsorption on *Al-S* /IL and its calcined forms (*Al-S* /IL400,* Al-S* /IL600 and *Al-S* /IL800).

Adsorbent	T (K)	Ln K_L_	ΔG (kJ mol^-1^)	ΔH (kJ mol^-1^)	ΔS (J mol^-1^)	*R*^*2*^
**Synozol KHL Red**						
*Al-S* /IL	298	1.06	-2.63	17.96	-50.19	0.87
	313	1.12	-2.93			
	333	0.33	-0.93			
	353	0.07	-0.22			
*Al-S* /IL400	298	0.61	-1.51	9.605	-27.89	0.89
	313	0.21	-0.53			
	333	0.12	-0.36			
	353	-0.05	0.15			
*Al-S* /IL600	298	-0.28	0.70	37.82	-130.1	0.91
	313	-1.43	3.73			
	333	-1.60	4.46			
	353	-2.91	8.56			
*Al-S* /IL800	298	-1.09	2.72	44.24	-157.3	0.99
	313	-1.91	4.98			
	333	-2.79	7.73			
	353	-3.93	11.53			
**Synozol KHL Blue**						
*Al-S* /IL	298	3.4050	-8.43	59.43	-178.9	0.79
	313	0.3754	-0.97			
	333	-0.2089	0.57			
	353	-0.6661	1.95			
*Al-S* /IL400	298	4.252	-10.53	632.8	-179.4	0.91
	313	2.6198	-6.81			
	333	0.5277	-1.4611			
	353	0.4891	-1.4356			
*Al-S* /IL600	298	0.4264	-1.056	38.36	-124.5	0.98
	313	-0.2455	0.639			
	333	-0.9194	2.545			
	353	-2.0592	6.044			
*Al-S* /IL800	298	-1.40	3.478	34.48	-125.8	0.95
	313	-1.57	4.09			
	333	-2.77	7.67			
	353	-3.42	10.04			

Generally, the negative values of ΔG for most adsorbents toward Synozol KHL Red at lower temperatures confirm that the adsorption process is spontaneous under these conditions. However, the gradual increase in ΔG values with increasing temperature, and their transition to positive values for some adsorbents, indicates that the adsorption process becomes less thermodynamically favorable at elevated temperatures, demonstrating that the spontaneity of adsorption is temperature dependent. The positive values of the enthalpy change (ΔH) confirm the endothermic nature of the adsorption process, particularly for Al-S/IL600 and Al-S/IL800, indicating that higher temperatures facilitate dye adsorption. Furthermore, the negative entropy change (ΔS) values suggest a decrease in randomness at the solid–solution interface during adsorption, reflecting the formation of a more ordered arrangement of dye molecules on the adsorbent surface as equilibrium is established^82–84^.

According to the data displayed for Synozol KHL Blue, although ΔG values were negative at 298 K, they turned positive at higher temperatures for several adsorbents, reflecting that the adsorption is non-spontaneous under elevated thermal conditions. High ΔH values, especially for *Al-S* /IL400, reflect the endothermic nature of the process. The consistently negative ΔS values across all adsorbents indicate a decrease in randomness, suggesting more organized interaction between dye molecules and adsorbent surfaces. These trends are in agreement with previous studies on structurally similar dyes such as Acid Blue 25, where ΔG was found negative (− 1.74 kJ mol)^-1^, ΔH positive (14.78 kJ mol)^-1^, and ΔS positive (55.41 J mol·K)^-1^, confirming spontaneous, endothermic adsorption accompanied by increased entropy at the interface^85^.

Hence, the thermodynamic parameters reveal that the adsorption mechanism is governed by the combined influence of enthalpy and entropy changes. Although adsorption is spontaneous under specific temperature conditions, the increase in ΔG with temperature for some adsorbents indicates that the feasibility of adsorption becomes increasingly dependent on the operating conditions. Therefore, the adsorption process exhibits condition-dependent spontaneity, consistent with the observed experimental performance. The obtained thermodynamic parameters also suggest that the adsorption mechanism involves both physical interactions and specific surface interactions between the dye molecules and the functional groups introduced by ionic liquid modification.

#### Adsorption kinetics

The adsorption kinetic models assess both the magnitude and mechanism of the proposed system adsorption for real scale application. The three common kinetic models (i.e. pseudo-first-order, pseudo-second-order models and Bangham’s model) were studied to assess the adsorption kinetics of Synozol KHL Red and Blue dyes onto the IL-conditioned alum sludge (*Al-S* /IL) and its calcined forms derivatives (400 °C, 600 °C, and 800 °C).

The calculated kinetic parameters are listed in Table 6. From the data, it is evident that the correlation coefficients (*R*^*2*^) obtained for the pseudo-second-order model were consistently higher than those for the pseudo-first-order model across all adsorbents and dyes. For instance, in the case of Synozol KHL Red, the *R*^*2*^ values for pseudo-second-order ranged from 0.948 to 0.993, while for pseudo-first-order they were lower (0.90 to 0.99). A similar trend was observed for Synozol KHL Blue, where *R*^*2*^ values exceeded 0.98 for pseudo-second-order and remained relatively low for pseudo-first-order.

**Table 6:** List of parameter obtained from kinetic models for dye adsorption by *Al-S* /IL and its calcined forms (*Al-S* /IL400,* Al-S* /IL600 and *Al-S* /IL800).

Adsorbent	Pseudo 1^st^-order			Pseudo 2^nd^-order				Bangham’s				
	Qe (mg g^-1^)	K_1_ (min^-1^)	*R*^*2*^	qe (mg g^-1^)		K_2_ (min^-1^)	*R*^*2*^	α		K_B_		*R*^*2*^
**Synozol KHL Red**												
*Al-S* /IL	1.132	0.0046	0.9		22.32	0.0016	0.99		0.7749	0.1488	0.96	
*Al-S* /IL400	1.261	0.0064	0.92		19.41	0.0015	0.96		0.7926	0.1383	0.97	
*Al-S* /IL600	1.115	0.0175	0.91		15.47	0.0010	0.95		0.808	0.1383	0.91	
*Al-S* /IL800	0.8970	0.0090	0.99		10.17	0.0011	0.97		0.8862	0.0756	0.93	
**Synozol KHL Blue**												
*Al-S* /IL	1.153	0.0028	0.82		17.07	0.0016	0.99		0.2465	0.0038	0.98	
*Al-S* /IL400	1.1272	0.0074	0.88		21.46	0.0017	0.99		0.1692	0.0064	0.91	
*Al-S* /IL600	0.944	0.0044	0.73		10.41	0.0046	0.99		0.2083	0.0029	0.95	
*Al-S* /IL800	0.5341	0.0037	0.84		3.962	0.0056	0.95		0.3107	0.0005	0.86	

Additionally, the calculated adsorption capacities (qe) of the pseudo-second-order model were in much better agreement with the experimental values, compared to the pseudo-first-order model. Such data clearly demonstrate that the pseudo-second-order model more accurately describes the kinetic behaviour of dye adsorption onto *Al-S* /IL materials. These findings suggest that chemisorption involving valence forces through sharing or exchange of electrons between the dye molecules and the adsorbent surface is the likely rate-determining step. These results align with the studies by^86^, who reported that pseudo-second-order kinetics governs the dye adsorption process onto modified alum-based adsorbents. Similarly^87^, emphasized that such a model is suitable for describing adsorption systems where chemical interactions dominate over physical ones. Furthermore, the Bangham’s model, although included for supplementary insight, showed relatively lower *R*^*2*^ values compared to the pseudo-second-order model, confirming that film diffusion is not the primary mechanism. Hence, it can be concluded that the adsorption of Synozol KHL Red and Blue dyes onto *Al-S* /IL and calcined forms follows pseudo-second-order kinetics, indicating chemisorption as the dominant mechanism, with the extent of adsorption being significantly influenced by the material’s surface properties and thermal treatment.

#### Stability and reuse facility

**Fig. 14:**
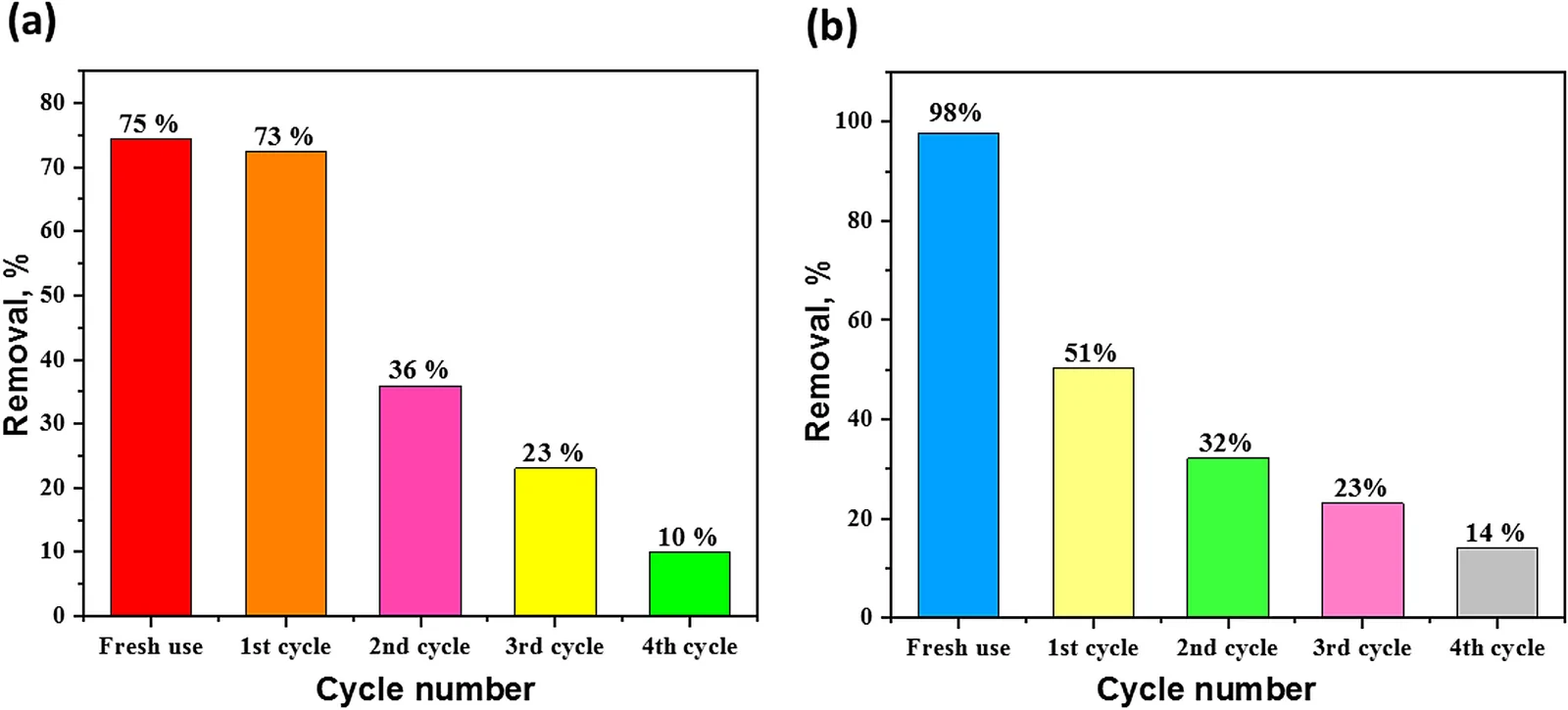
Reuse performance of the *Al-S* adsorbent for (**a**) Synozol KHL Red and (**b**) Synozol KHL Blue.

For the purpose of evaluating the sustainability opportunity of the suggested adsorbent, the material reusability was evaluated. The adsorbent is collected, after the experiment is carried out, by filtration and then subjected to successive washing with distilled water. Subsequently, the cleaned catalyst is oven dried at 150 °C in an electrical furnace for 1 h. For the same adsorption isotherm time the data is compared with the fresh adsorbent use and displayed in Fig. 14.

According to the data displayed in Fig. 14 (a) for the removal efficiency for Synozol KHL Red revealed that the dye remained relatively high during the first cycle, with only a slight decline compared to the fresh adsorbent (75% removal) that reduced to 73% removal. However, a gradual decrease was observed, reaching its lowest value in the fourth cycle reached only 10% removal. In contrast, Fig. 14 (b) demonstrates a more pronounced reduction in removal efficiency for Synozol KHL Blue after each reuse, with a sharp drop after the fresh use declined to 51% removal, suggesting a faster loss of available active sites.

It is noteworthy to mention that the progressive decline in performance for both dyes can be attributed to partial blockage of adsorption sites, structural fatigue of the adsorbent surface, and possible irreversible binding of dye molecules during repeated adsorption–desorption processes. Nevertheless, the retention of a considerable portion of adsorption capacity after multiple cycles highlights the stability of the *Al-S /IL* system and its suitability for repeated wastewater treatment applications. Such reusability not only reduces the operational cost but also aligns with the principles of sustainable and eco-friendly water remediation^62^.

#### Comparison of data with literature

Based on the current experimental work data, some previously stated related research cited in the published articles are investigated and compared with the present data. The comparative investigation data are summarized in Table 7 with various treatment types. The comparison is based on using modified low-cost adsorbents that reported in literature with the present study. The data displayed in Table 7 revealed that the calcined IL-conditioned alum sludge at 400°C referred as *Al-S /*IL400 demonstrated a high adsorption capacity of Synozol KHL Blue recorded as 90.1mg g^-1^, while the alum sludge conditioned by ionic liquid (*Al-S* /IL) tabulated 43.6 mg g^-1^ for Synozol KHL Red. Although other systems including Mg Fe_2_O_4_ SiC, CuZn Fe_2_O_4_ biochar, and CdAg Ferrite AS400 Ferrite–CdAg achieved higher capacities for various dyes or heavy metals, some challenges are related to such materials such as the separation technique after treatment, high synthesis cost, or operational complexity. Also, it is noteworthy to mention that the currently proposed material verifies the sustainability basis.

**Table 7:** Comparison of different wastewater treatment low cost adsorbents for treating various types of wastewater.

Adsorbent	Adsorbate	Operating parameters	Adsorption capacity	Ref
Condtioned alum sludge (calcined form 400) (*Al-S* /IL400)	Synozol KHL Blue dye	Dose 1 g L^-1^ ;pH 7; 20ᵒc; 100 ppm; 24 h	90.1 mg g^-1^	Current work
Conditioned alum sludge (*Al-S* /IL)	Synozol KHL RED dye	Dose 1 g L^-1^; pH 6.3; 20ᵒc; 30 ppm ; 5 h	43.6 mg g^-1^	Current work
Mg Fe_2_O_4_–SiC composite	Azo dye direct black BN dye	1.5 g L^-1^; 20 ppm; 120 min	84.637 mg g^-1^	^67^
Carbon/cobalt ferrite (C/CFO) nanocomposite	Methylene Blue dye	0.6 g L^-1^; 50 mg L^**-1**^	43.05 mg g^-1^	^88^
CuZnFe_2_O_4_–biochar composite	BPA	20 mg L^-1^; 60 min	101.5 mg g^-1^	^89^
CuZn Fe_2_O_4_–biochar composite	sulfamethoxazole (SMX)	20 mg L^-1^	99.99 mg g^-1^	^89^
Sawdust/magnetite composite (SF-(2:1))	Synozol Red KHL dye	1 g L^-1^; 20 ppm; 5 min	21.71 mg g^-1^	^73^
Fly ash/Ni Fe_2_O_4_ composites	Congo red dye	0.1 g 100 Ml^**-1**^; 25 mg L^-1^; 180 min	23.33 mg g^**-1**^	^90^
Ni Fe_2_O_4_ nanocomposite activated carbon	Malachite green dye	0.5 g; 50 mg L^-1^; 60 min	83 mg g^-1^	^91^
Co Fe_2_O_4_–chitosan composite	Methyl Orange (MO)	240 min	66.18 mg g^-1^	^92^
CdAgF (Ferrite AS400F(Ferrite)-CdAg	Synozol Red KHL dye	1 g L^-1^; 20 ppm; 60 min	149.031 mg g^-1^	^27^
CdAg Ferrite AS40 (Ferrite -CdAg	Synozol Red KHL dye	1 g L^-1^; 20 ppm ; 20 ppm	102.56 mg g^-1^	^27^
Sawdust/magnetite	Red KHL dye	pH 3.0; dose 1 g L^-1^	21.71 mg g^-1^	^93^
Water Treatment plant (WTP) Abbanoa industry, Truncu Reale, Italy	Cd(II)	pH 4.5; 25 °C; 0.1 g; 25 mL; 24 h	0.040 mmol Cd(II) g^-1^	^94^
Water Treatment plant (WTP) Abbanoa industry, Truncu Reale, Italy	Zn(II)	pH 4.5; 25 °C; 0.1 g; 25 mL; 24 h	0.050 mmol Zn(II) g^-1^	^94^
NA	Acid Blue 25	Co 500 mg L^-1^; pH 4; 120 min	59 mg g^-1^	^95^
Shahid Beheshti WTP, Iran	Humic acid	pH 5.56; AS 19.71 g L-1; Humic acid 12.28 mg L^-1^	0.47 mg g^-1^	^96^
Rice husk carbon	Acid yellow 36 dye	pH 3.0; 30 °C; 180 min	86.9 mg g^-1^	^97^
Orange peel	Rhodamine B dye	Co 120 mg L^-1^; dose 1 g L-1; pH 11; 120 min	2.56 mg g^-1^	^98^
Water supply plant, Nanjing, China	Phosphorus	AS 0.5 g; pH 7.0; 20 °C; 24 h; 100 mL	0.90 mg g^-1^	^99^
Water supply plant, Shebin El Kom, Egypt	Phenol	AS 2 g; Co 100–1000 mg L^-1^; 298 K; 1 h	275 mg g^-1^	^75^
Southern Shebin WTP, Egypt	Procion Blue dye	AS 2 g; pH 7; Co 11.8 mg L^-1^; 298 K; 1 h	6.5 mg g^-1^	^100^
Rice husk	Brilliant green dye	Co 100 mg L^-1^; dose 1 g L-1; pH 10	66.0 mg g^-1^	^101^

In contrast, magnetically enhanced materials like sawdust/magnetite, despite moderate uptake (~ 21.7 mg g^-1^), enable easy magnetic recovery, minimizing secondary pollution and allowing reuse. The current IL-conditioned alum sludge system combines high efficiency, environmental safety, and reusability, making it a sustainable option for textile dye removal since it depends on managing waste stream depending on end waste criteria.

## Conclusion

Ionic liquid was introduced and applied for alum sludge conditioning as a green conditioner for improving alum sludge dewaterability effect. The optimal operational variables were assessed and recorded at one minute of dewatering time and pH 3.0 that showed 47% dewatering performance. Further confirmation data demonstrated the change in particle size of the sludge through its morphology change after conditioning and the reduction in zeta potential signifies the elevation in the flocculation ability. Also, the data compared with the traditional conditioners and comparison with the commercial conditioners such as polyelectrolytes and surfactant was proposed and the results exhibited the importance of the current study in improving the sludge dewaterability with an environmentally friendly conditioner. All the experimental data is confirmed with the characterization of the sludge prior and after conditioning after and prior to the addition of chemical conditioners. Also, the green ionic liquid conditioning is compared with the available conditioners in literature and the results demonstrated that the ionic liquid could be studied as an ideal alternative of available commercial conditioners. To assure the sustainable affinity the dewatered sludge is exposed for dye removal from wastewater as a win–win situation using adsorption technique. The results showed that *Al-S*/IL and *Al-S*/IL400 exhibited the highest adsorption capacities reached 43.6 to 90.1 mg g^-1^ for Synozol KHL Red and Blue, respectively at the optimal operating condition of adsorbent is 0.5 g L^-1^, 30 ppm of initial dye concentration, pH 6.3 at room temperature for Synozol KHL Red and at the operating condition 1 g L^-1^ of adsorbent , 100 ppm of initial dye concentration, pH 7 at room temperature for Synozol KHL Blue dyes. The system is distinguished by its superior performance in sludge dewaterability enhancement and treating wastewater in an economic circular economy approach.

## Data Availability

The datasets used and/or analysed during the current study available from the corresponding author on reasonable request.
